# Host defence peptide plectasin targets bacterial cell wall precursor lipid II by a calcium-sensitive supramolecular mechanism

**DOI:** 10.1038/s41564-024-01696-9

**Published:** 2024-05-23

**Authors:** Shehrazade Jekhmane, Maik G. N. Derks, Sourav Maity, Cornelis J. Slingerland, Kamaleddin H. M. E. Tehrani, João Medeiros-Silva, Vicky Charitou, Danique Ammerlaan, Céline Fetz, Naomi A. Consoli, Rachel V. K. Cochrane, Eilidh J. Matheson, Mick van der Weijde, Barend O. W. Elenbaas, Francesca Lavore, Ruud Cox, Joseph H. Lorent, Marc Baldus, Markus Künzler, Moreno Lelli, Stephen A. Cochrane, Nathaniel I. Martin, Wouter H. Roos, Eefjan Breukink, Markus Weingarth

**Affiliations:** 1 NMR Spectroscopy, Bijvoet Centre for Biomolecular Research, Department of Chemistry, Utrecht University, Utrecht, The Netherlands; 2 Membrane Biochemistry and Biophysics, Department of Chemistry, Bijvoet Centre for Biomolecular Research, Utrecht University, Utrecht, The Netherlands; 3 Moleculaire Biofysica, Zernike Instituut, Rijksuniversiteit Groningen, Groningen, The Netherlands; 4 Biological Chemistry Group, Institute of Biology Leiden, Leiden University, Leiden, The Netherlands; 5 Department of Biology, Institute of Microbiology, ETH Zürich, Zürich, Switzerland; 6 Magnetic Resonance Center (CERM) and Department of Chemistry “Ugo Schiff”, University of Florence, Sesto Fiorentino, Italy; 7 Consorzio Interuniversitario Risonanze Magnetiche MetalloProteine (CIRMMP), Sesto Fiorentino, Italy; 8 School of Chemistry and Chemical Engineering, Queen’s University Belfast, Belfast, UK

**Keywords:** Solid-state NMR, Antibiotics, Atomic force microscopy, Mechanism of action

## Abstract

Antimicrobial resistance is a leading cause of mortality, calling for the development of new antibiotics. The fungal antibiotic plectasin is a eukaryotic host defence peptide that blocks bacterial cell wall synthesis. Here, using a combination of solid-state nuclear magnetic resonance, atomic force microscopy and activity assays, we show that plectasin uses a calcium-sensitive supramolecular killing mechanism. Efficient and selective binding of the target lipid II, a cell wall precursor with an irreplaceable pyrophosphate, is achieved by the oligomerization of plectasin into dense supra-structures that only form on bacterial membranes that comprise lipid II. Oligomerization and target binding of plectasin are interdependent and are enhanced by the coordination of calcium ions to plectasin’s prominent anionic patch, causing allosteric changes that markedly improve the activity of the antibiotic. Structural knowledge of how host defence peptides impair cell wall synthesis will likely enable the development of superior drug candidates.

## Abstract (web summary)

Plectasin, a natural host defence peptide of fungal origin, uses a calcium-sensitive supramolecular killing mechanism to block bacterial cell wall synthesis.

## Main

The rise of multidrug-resistant bacteria is a severe threat to human health and calls for the development of antibiotics that use new mechanisms^1–3^.

The discovery of the antibiotic plectasin, a host defence peptide isolated from the fungus *Pseudoplectania nigrella*, aroused strong interest^4,5^. Plectasin adopts a compact cysteine-stabilized fold that is representative of a large family of host defence peptides in invertebrates^4^. The peptide and its variants^6–9^ show high activity against pathogens such as methicillin-resistant *Staphylococcus aureus*, *Streptococcus pneumoniae* or *Mycobacterium tuberculosis*, including in animal infection models^4,10^. Plectasin exerts its bactericidal activity by targeting the peptidoglycan precursor lipid II^5^ in the plasma membrane, blocking the cell wall biosynthesis.

**Fig. 1:**
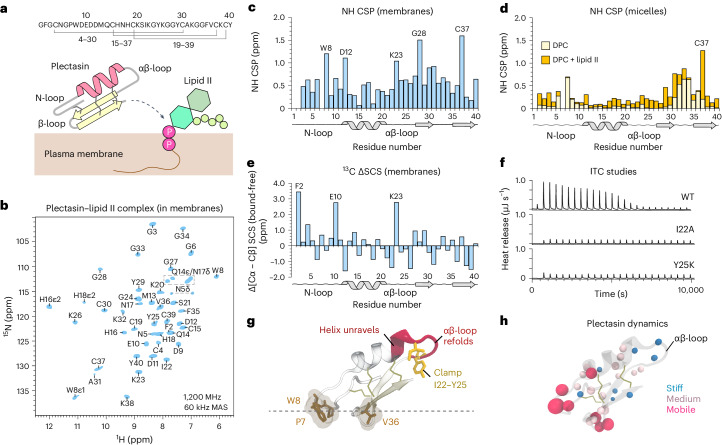
Plectasin’s target binding in membranes. **a**, Plectasin targets the peptidoglycan precursor lipid II in the bacterial plasma membrane. The amino acid sequence is shown, including the topology of the three disulfide bridges. **b**, 2D ^15^N-^1^H (NH) ssNMR spectrum of lipid II-bound plectasin in membranes. **c**, CSPs of lipid II-bound plectasin in membranes compared to unbound plectasin. Source data are provided. **d**, CSPs of plectasin in micelles in the presence of dodecyl phosphocholine (DPC) (light yellow) and DPC plus lipid II (dark yellow), compared to unbound plectasin. Data replotted from ref. ^5^. Source data are provided. **e**, ^13^C (CαCβ) secondary chemical shift^14^ differences comparing unbound and lipid II-bound plectasin in membranes (Extended Data Fig. 1e). Source data are provided. SCS, secondary chemical shift. **f**, Representative ITC data (acquired in triplicate) show that plectasin mutants I22A and Y25K do not bind lipid II. **g**, Upon lipid II binding, plectasin’s αβ-loop changes its conformation, stabilized by a clamp-like interaction of I22 and Y25. **h**, ssNMR ^15^N *T*_1ρ_ dynamics show that the long αβ-loop of plectasin is rigid in the lipid II-bound state. The size of the spheres represents the dynamics per residue. Source data

Despite the prominence of plectasin, its mechanism is insufficiently understood. The only available molecular mechanistic data were obtained using detergent micelles^5^, suggesting a conventional one-drug/one-target complex (Fig. 1a). However, this binding mode appears incomplete considering recent studies that show that lipid II binders such as teixobactin^11,12^ and clovibactin^13^ use supramolecular mechanisms on the membrane surface to kill bacteria. What is more, the previously suggested complex interface^5^, in which plectasin uses its β-sheet to target lipid II, cannot fully explain functional observations. Here, the most striking observation is the small level of agreement between mutations that improve plectasin’s activity^6–10^ and residues that were identified in micelles^5^ as relevant for lipid II binding. In addition, plectasin contains a conspicuous anionic patch of four consecutive negatively charged amino acid residues (9-DEDD-12) to which no function could be assigned. Together, this indicates that important aspects of plectasin’s action remain elusive.

In this Article, we report the mode of action of plectasin in biological membranes at several length scales. We show that plectasin uses a new type of supramolecular antimicrobial action that is critically enhanced by the binding of calcium ions to the anionic patch.

## Results

### Target binding in membranes

**Extended Data Fig. 1:**
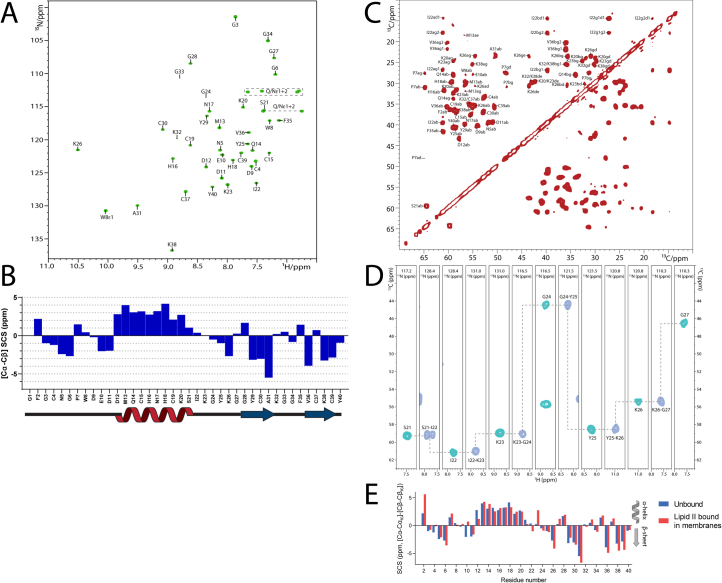
Solution state NMR of soluble plectasin and solid-state NMR of plectasin bound to Lipid II in membranes. **A)** Solution state ^1^H^15^N-HSQC spectrum of unbound plectasin. Spectrum was acquired at 900 MHz (^1^H frequency) at 295 K. Chemical shifts match published data^1,2^. **B)** Calculated secondary chemical shifts^3^ of unbound plectasin in solution, showing the characteristic α-helix, β-sheet fold of CS-αβ defensins. Additionally, the N-terminal loop tends toward a β-strand fold. Source data are provided. **C**) 2D PARISxy^4^ ^13^C^13^C spectrum of plectasin in complex with Lipid II, acquired at 950 MHz (^1^H frequency), 18 kHz MAS, and 270 K sample temperature with a ^13^C-^13^C mixing time of 20 ms. **D)** Strip plots of the 3D experiments showing backbone assignments of the αβ-loop from S21-G27. Strip plots show slices of ^1^H-detected solid-state 3D CαNH (cyan) and Cα(CO)NH (blue) ssNMR experiments. **E)** Comparison of Cαβ secondary chemical shifts (SCS^3^) of plectasin in the free, soluble state (blue) and in the Lipid II-bound in membranes (red). The difference of the SCS is shown in Fig. 1e of the main text. Source data are provided. Source data

To study plectasin’s mechanism in a relevant environment, we used solid-state nuclear magnetic resonance (ssNMR), a non-invasive technique that allows for studies of antibiotics in biological membranes at high resolution^11^. We produced uniformly ^13^C,^15^N-labelled plectasin in *Escherichia coli* SHuffle cells to ensure correct disulfide-bond topology. Solution-state NMR and activity studies confirmed the identity^4^ of plectasin (Table 1, Extended Data Fig. 1a,b and Supplementary Table 1). Next, we co-assembled ^13^C,^15^N-plectasin and lipid II in membranes and acquired high-quality ssNMR spectra (Fig. 1b) that show the formation of a well-defined complex.

**Table 1:** Antimicrobial activity assays (under standard conditions) of plectasin mutants (MIC in μg ml^−1^)

Drug/strain	*S. simulans 22*
Plectasin wt	0.78
I22D	>50
I22F	>50
I22K	>50
I22L	1.56
I22V	1.56
K23A	6.25
Y25F	1.56
Y25H	≤0.78
Y25I	6.25
Y25L	3.13
Y25V	6.25
K26A	3.13
K26R	≤0.78
Y29A	12.5
Y29F	1.56
Y29R	1.56
Y40R	>50
Y40F	1.56
Vancomycin	0.78

See Supplementary Table 1 for additional activity assays.

We fully assigned the chemical shifts of lipid II-bound plectasin in membranes (Extended Data Fig. 1c,d). NMR signal changes, so-called chemical shift perturbations (CSPs), between free and bound plectasin report on structural changes upon complex formation. In micelles, only minor, local signal changes in plectasin’s β-sheet could be observed (Fig. 1d)^5^. By contrast, in membranes, we observed marked signal changes in the entire peptide upon lipid II binding (Fig. 1c,e). We measured strong signal changes not only in the β-sheet, which gets stabilized in the complex, but also in the αβ-loop that connects plectasin’s helix and β-sheet and in the long N-terminal loop (N-loop) around the anionic patch (D9, E10, D11, D12). As no functions were hitherto attributed to the αβ-loop or anionic patch, these observations surprised and insinuated that plectasin’s action is more complex than previously reported^5^. Secondary chemical shift^14^ changes further show that the α-helix unravels at its C-terminus (S21, I22) upon lipid II binding, extending the αβ-loop by two residues (S21 and G27) (Fig. 1e and Extended Data Fig. 1e).

**Extended Data Fig. 2:**
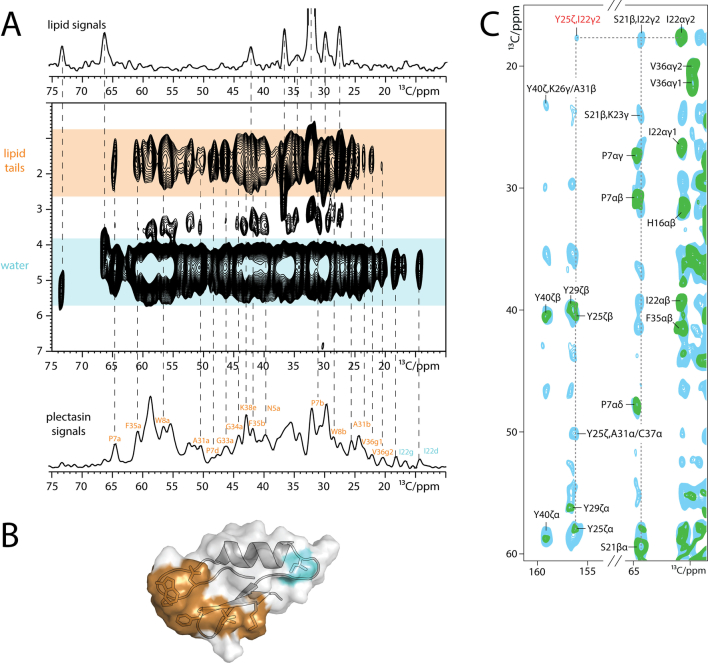
Hydrophobic residues of the β-loop and the N-loop anchor plectasin in the membrane. **A,B)** T_2_-edited ^1^H(^1^H)^13^C experiments^5^ of ^13^C^15^N-plectasin in complex with Lipid II probing membrane insertion. In this experiment, after a T_2_-filter that destroys magnetization on rigid moieties, magnetization from mobile water and lipid molecules is transferred to the rigid antibiotic using ^1^H^1^H mixing, and eventually read out on ^13^C using a short cross-polarization. **A)** Top: 1D spectrum displaying mobile lipid resonances after dephasing of rigid plectasin resonances using a T_2_-filter of 2.5 ms and no ^1^H^1^H mixing. Middle: T_2_-edited 2D ^1^H(^1^H)^13^C experiment with a T_2_-filter of 2.5 ms and ^1^H^1^H mixing time of 5 ms. Bottom: 1D ^13^C CP spectrum of the complex. **B)** ssNMR-derived membrane topology mapped onto plectasin. Residues that help to anchor plectasin in the membrane are highlighted in brown colors, the water-exposed Ile22 is colored in blue. **C**) ssNMR contact between I22 and Y25. Overlay of two 2D ^13^C^13^C PARIS-xy^4^ spectra of ^13^C^15^N-plectasin in complex with lipid II in membranes with either a short (62 ms, green) or long (600 ms, blue) mixing time. Spectra were recorded at a magnetic field of 950 MHz (^1^H-frequency), a sample temperature of 270 K and 18 kHz MAS.

**Extended Data Fig. 3:**
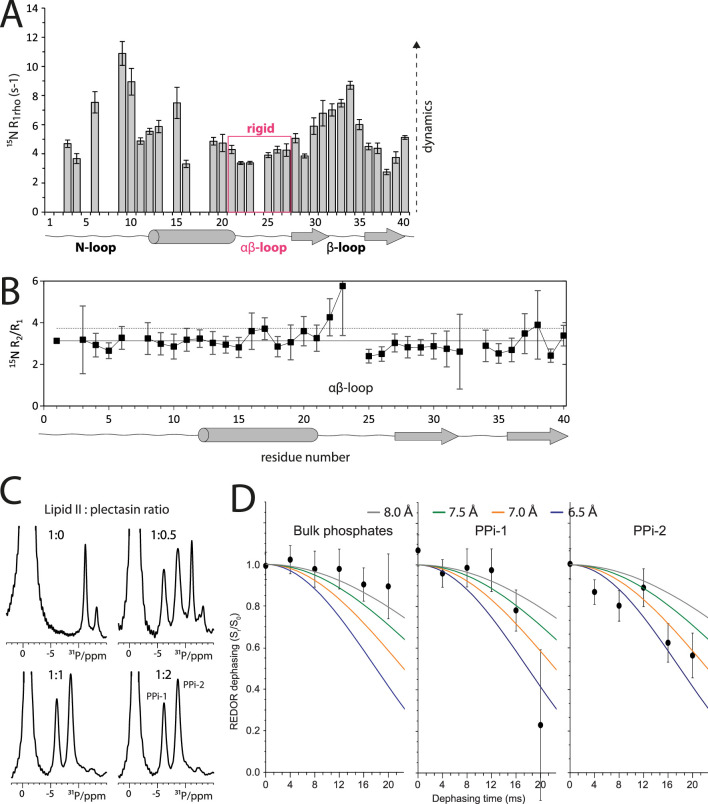
NMR dynamics of Lipid II-bound and free plectasin, and investigation of the plectasin – pyrophosphate interaction. **A)** ssNMR ^15^N-R_1ρ_ measurement of plectasin in complex with Lipid II in membranes, derived from ^1^H-detected data^6^. ssNMR spectra were recorded at 800 MHz (^1^H frequency), 60 kHz MAS, and 305 K sample temperature. A ^15^N spin lock amplitude of 18 kHz and with durations of 0, 5, 10, 20, 40, 80 and 120 ms were applied. The heights of amide resonances were fit using a single exponential decay. The error bars show the standard error of the fit. Source data are provided. **B)** Backbone amide dynamics determined with solution NMR on free plectasin. Measurements were done at 600 MHz (^1^H frequency) and 298 K. Experiment details are provided in the methods section. The increase in the ratio of ^15^N R_2_/R_1_ points to slow conformational exchange dynamics of the αβ-loop in the free state. This means that, in the absence of Lipid II, the αβ-loop is plastic, sampling different conformations. Source data are provided. **C,D)** ^31^P ssNMR experiments of the plectasin – lipid II complex. **C)** ^31^P CP spectra of Lipid II in DOPC membranes titrated with plectasin. Spectra were acquired at 500 MHz (^1^H-frequency), a sample temperature of 270 K and 10 kHz MAS for spectrum in the absence of plectasin and 13.5 kHz MAS for the other spectra. **D)** ssNMR ^31^P{^13^C}-REDOR experiments show that plectasin directly interacts with the pyrophosphate of Lipid II. REDOR experiments were conducted at 18.8 T (800 MHz ^1^H frequency) with 60 kHz MAS and a sample temperature of 290 K. ^31^P resonances of the lipid II pyrophosphate dephase upon reintroduction of ^13^C-^31^P dipolar couplings, while the bulk phosphate remains largely unaffected. 1D REDOR spectra were deconvoluted with Lorentzian and Gaussian functions. We fitted three peaks at –0.31 ppm (bulk phosphate), –5.76 ppm (named PPi-1), and –8.12 ppm (named PPi-2) with Lorentzian functions. An additional broad peak at –10.23 ppm, due to little ^31^P additional species not fully characterized, was fitted with a gaussian function to improve the quality of the PPi-2 integral. The signal integration was performed with trapezoidal method with the function numpy.trapz from numpy on the deconvoluted peaks. To fit the experimental spectra in the deconvolution procedure, the least-square method was used. Source data are provided. The deconvolution software for fitting and analyzing the data are written using Python 3.9.12^7^, using the libraries numpy^8^ 1.23.3. nmrglue^9^ 0.9. matplotlib^10^ 3.5.2. lmfit^11^ 1.0.3. The package is available upon request to the authors. Dephasing curves are superimposed over simulated REDOR profiles corresponding to a ^31^P-^13^C distance of 8.0 Å (grey), 7.5 Å (green), 7.0 Å (orange), and 6.5 Å (blue), corresponding to ^31^P-^13^C dipolar couplings of –23.9 Hz, –29.0 Hz, –35.7 Hz and –44.6 Hz, respectively. Source data

We used mutagenesis in combination with functional studies to explore the role of the αβ-loop for plectasin’s binding mechanism. Strikingly, mutants of αβ-loop residues I22 and Y25 showed weak or no activity toward several isolates of Gram-positive bacteria (Table 1 and Supplementary Table 1). Isothermal titration calorimetry (ITC) data show that the loss of antimicrobial activity is caused by a complete loss of binding affinity to lipid II (Fig. 1f). We surmised that the long hydrophobic and aromatic residues (I22, Y25) could be necessary to anchor plectasin to the membrane. However, ssNMR experiments that probe the interactions of plectasin with lipids or water^8^ show that I22 and Y25 are not in contact with the membrane, ruling out that the αβ-loop acts as a membrane anchor (Extended Data Fig. 2a,b). Rather, our data show that the hinge of the N-loop (N5, P7, W8) and the β-loop/β-sheet region (Y29, A31, F35, V36) partition into the bilayer (Fig. 1g). Thus, the αβ-loop may instead be involved in lipid II binding. To test this possibility, we acquired two-dimensional (2D) ^13^C-^13^C (CC) ssNMR data and observed unambiguous contacts between the long hydrophobic side chains of I22 and Y25 (Extended Data Fig. 2c), suggesting that these residues form a clamp-like interaction that locks the αβ-loop in a primed conformation that is decisive for plectasin’s activity (Fig. 1g). This conclusion is corroborated by ssNMR relaxation data^15^ that show an astonishing rigidity for the long αβ-loop in the complex, whereas the N-loop and β-loop remain highly mobile (Fig. 1h and Extended Data Fig. 3a,b), and it also aligns with mutagenesis data that show that hydrophobic or aromatic residues at positions 22 and 25 are required for activity (Table 1 and Supplementary Table 1).

### The complex interface

**Fig. 2:**
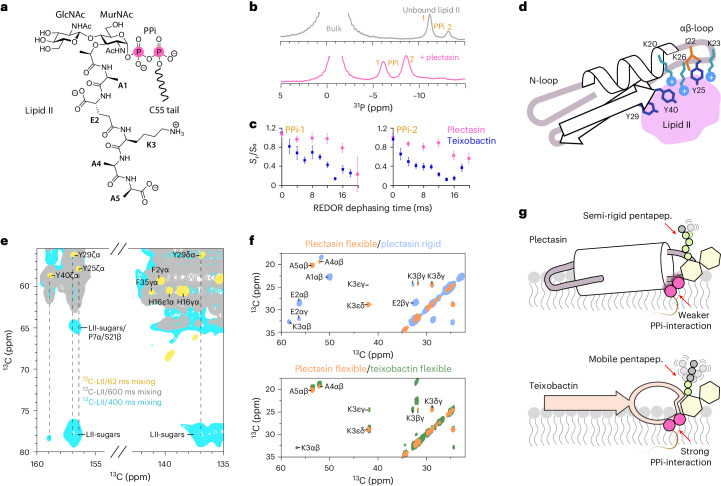
Lipid II binding interface. **a**, Chemical structure of lipid II. **b**, 1D ^31^P ssNMR data show strong shifts of the lipid II pyrophosphate signals upon binding of plectasin. **c**, ^13^C-^31^P ssNMR REDOR^16^ data show slower decay of the pyrophosphate signals in the ^13^C-plectasin–lipid II complex compared to the ^13^C-teixobactin–lipid II complex. This implies that the interaction between PPi and plectasin is more dynamic. The reported error is one unit of standard deviation, calculated on the *S*_r_/*S*_0_ ratio on the bases of the experimental errors in the integrated spectra. Source data are provided. **d**, Cartoon representation of plectasin showing the presumed residues that interact with lipid II. **e**, Overlay of 2D ssNMR ^13^C^13^C PARISxy^45^ spectra of plectasin in complex with NMR-visible ^13^C,^15^N-labelled lipid II (in cyan) and NMR-invisible ^12^C,^14^N lipid II (in yellow and grey) with different CC mixing times that show contacts between aromatic residues (Y25, Y29, Y40) of plectasin and lipid II sugars. LII, lipid II. **f**, A combination of scalar^17^ (that show only mobile residues) and dipolar (that show only rigid residues) 2D CC ssNMR spectra show that the pentapeptide is more flexible in the teixobactin–lipid II complex than in the plectasin–lipid II complex. Upper panel: overlay of scalar (in orange) and dipolar (in blue) ssNMR spectra of the pentapeptide in the plectasin–lipid II complex. Lower panel: overlay of scalar ssNMR spectra of the pentapeptide in complex with plectasin (in orange) and teixobactin (in green). **g**, Cartoon representations of the plectasin–lipid II and teixobactin–lipid II complexes. Source data

Plectasin targets the cell wall precursor lipid II, a complex C55-polyisoprenoid-based lipid with a conserved pyrophosphate (PPi) group, a headgroup composed of the sugars MurNAc and GlcNAc and a pentapeptide (Fig. 2a).

First, we investigated how plectasin targets the lipid II–PPi group in membranes. We acquired 1D ^31^P ssNMR spectra that show strong shifts of the PPi signals upon addition of plectasin, suggesting a direct coordination (Fig. 2b). After addition of 1 equivalent of plectasin, the unbound PPi peaks disappeared, indicative of the formation of an equimolar plectasin–lipid II complex (Extended Data Fig. 3c). Using ^13^C-^31^P rotational-echo double-resonance (REDOR)^16^ ssNMR experiments that measure the proximity between ^13^C-labelled plectasin and PPi, we show that plectasin directly interacts with the PPi group (Extended Data Fig. 3d). However, unlike we previously reported for teixobactin^11^, we did not observe signals in 2D ^1^H-^31^P ssNMR data for the plectasin–lipid II complex, suggesting that the plectasin–lipid II interface is rather dynamic. The enhanced dynamics agrees with comparative ^13^C-^31^P ssNMR REDOR data that probe the strength of the interaction between the PPi group of lipid II and either ^13^C-plectasin or ^13^C-teixobactin (Fig. 2c). These data show that the teixobactin–PPi interaction is stronger compared to the plectasin–PPi interaction.

Structure–activity data (Fig. 1c,e,f,h) show the essential role of plectasin’s αβ-loop for lipid II binding and antibacterial activity. It hence appears likely that the αβ-loop region, with its numerous free hydrogen bonding opportunities and its cationic residues (K20, K23, K26), is directly involved in the coordination of anionic lipid II headgroup (Fig. 2d). Indeed, mutagenesis studies show a fivefold to tenfold reduction in activity for the plectasin mutants K23A and K26A (Table 1).

**Extended Data Fig. 4:**
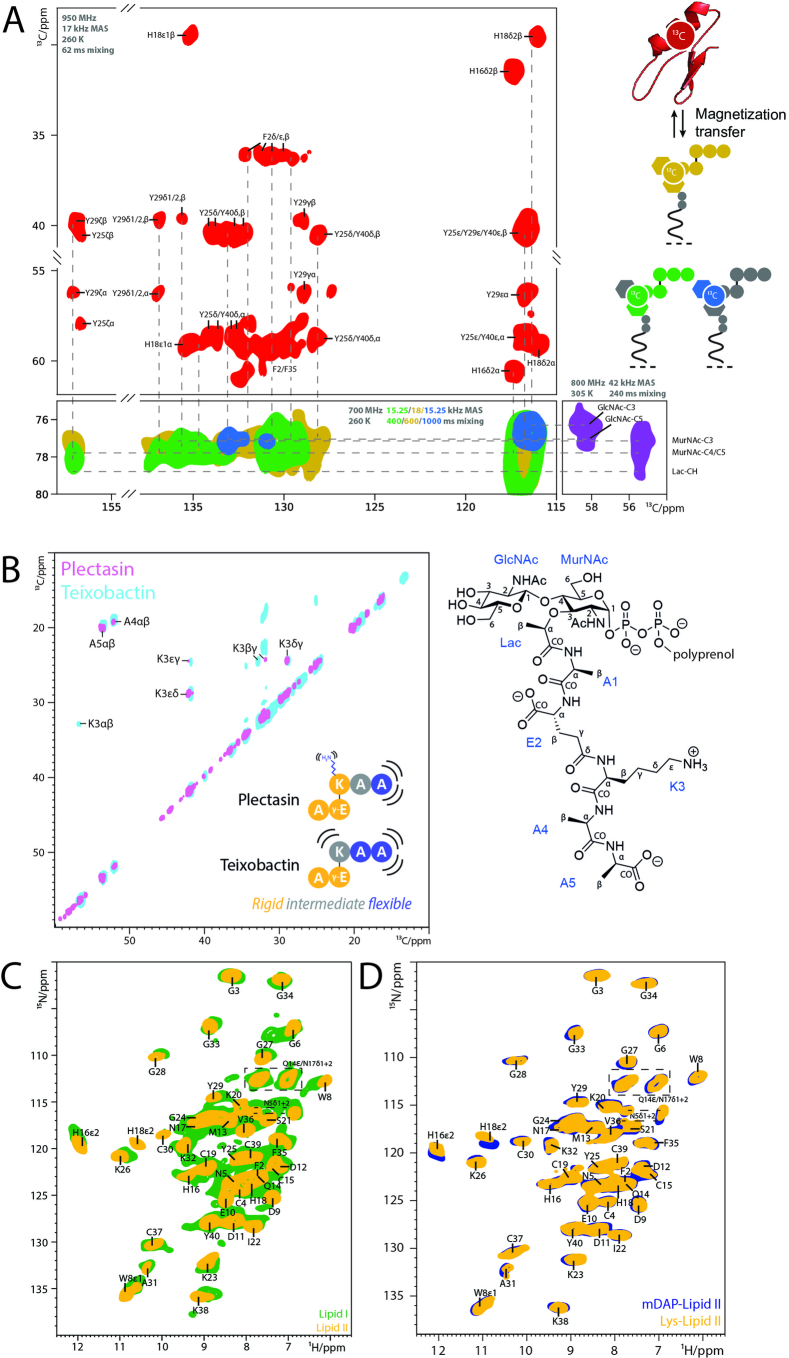
The MurNAc sugar is directly at the complex interface. ssNMR 2D ^13^C-^13^C spectra investigating the plectasin - Lipid II interface. **A)** 2D ^13^C-^13^C PARISxy^4^ spectra probing magnetization transfer between plectasin and lipid II. Top (in red): spectrum of plectasin in complex with unlabeled ^12^C-Lipid II that shows signals of plectasin’s aromatic sidechains. Bottom left: overlay of 2D PARISxy CC spectra recorded with ^13^C^15^N-plectasin and Lipid II with 3 different ^13^C^15^N-labelling schemes: in yellow with the full pentapeptide-MurNAc-GlcNAc Lipid II headgroup ^13^C^15^N-labelled, in green with only pentapeptide-MurNAc ^13^C^15^N-labelled, and in blue with only the GlcNAc ^13^C^15^N-labelled. Bottom right (in magenta): zoom in to the sugar region of 2D CC spectrum of the full Lipid II headgroup, acquired with unlabeled ^12^C-plectasin that shows the relevant sugar signals. We observed clear magnetization transfer between aromatics of plectasin and the sugars of lipid II, but spectral overlap between the MurNAc and GlcNAc sugars complicated spectral analysis. We therefore also recorded spectra with two different labeling schemes, where only one of the two sugars was ^13^C-labelled. Our data show that more of the interfacial plectasin – sugar signals relate to the MurNAc sugar that is covalently bound to the Lipid II PPi-group, while we could only observe weak magnetization transfer between plectasin and the GlcNAc sugar with a very long mixing time (1 s). **B)** Scalar-based ssNMR spectra exclusively show mobile residues and can, as we previously showed^12,13^, be used to resolve the involvement of the pentapeptide in the drug – Lipid II interface. The figure shows an overlay of scalar-based CC TOBSY spectra of ^13^C^15^N-lipid II in complex with plectasin (magenta) or teixobactin (blue). Spectra were acquired at 950 MHz (^1^H frequency) for plectasin and 700 MHz (^1^H frequency) for teixobactin, both at a sample temperature of 305 K and using 6 ms CC mixing time. The pentapeptide shows a higher rigidity in the plectasin – Lipid II complex as compared to the teixobactin – Lipid II complex. This suggests that plectasin directly binds to the pentapeptide as suggested previously in biochemical assays^14^, while teixobactin does not interact with the pentapeptide of lipid II^12,13^. Note that the teixobactin spectrum was previously published^13^. **C**) Overlay of 2D ssNMR NH spectra of ^15^N-plectasin in complex with Lys-Lipid II (yellow) and Lys-Lipid I (green), acquired in DOPC liposomes. Spectra with Lipid I are globally the same as for Lipid II, but more heterogeneous. The GlcNAc sugar (which lacks for Lipid I) must thus have some importance for the plectasin binding to Lipid II^2^, potentially by modulating the conformation of the MurNAc sugar or by contributing to the plectasin – Lipid II supra-structure, while its direct contribution to the interface is small. **D**) Overlay of 2D ssNMR NH spectra of ^15^N-plectasin in complex with Lys-Lipid II (yellow) and mDAP-Lipid II (blue). The third residue of the pentapeptide (lysine) is substituted by meso-diaminopimalic acid in mDAP-Lipid II. The spectra overlay well, demonstrating that the lysine-sidechain of the pentapeptide is not involved in the complex interface, which agrees with our analysis of the scalar spectrum shown in B).

To explore how the lipid II sugars contribute to the complex interface, we assembled a complex formed by ^13^C,^15^N-plectasin and ^13^C,^15^N-lipid II^12^ and acquired 2D CC ssNMR spectra. We could detect an unambiguous interfacial contact between lipid II sugars and the Y29 aromatic side chain of plectasin and a weaker unambiguous contact with the Y40 aromatic side chain, as well as an ambiguous contact with the Y25 side chain (Fig. 2e). Mutagenesis studies (Table 1 and Supplementary Table 1) confirmed that residues 25, 29 and 40 are of functional importance. Note that the number of interfacial contacts is relatively small due to spectral overlap and also due to the above-observed dynamics of the complex interface. Residues Y25, Y29 and Y40 are co-localized at the β-sheet–αβ-loop junction and hence point towards a molecular region that coordinates lipid II (Fig. 2e). To pinpoint the role of the lipid II sugars, we also conducted analogous ssNMR experiments with lipid II molecules that were specifically ^13^C,^15^N-labelled at either the GlcNAc or the MurNAc sugar (Extended Data Fig. 4a and Supplementary Fig. 1). Here, only the MurNAc sugar showed sizeable interfacial contacts, which demonstrates that MurNAc, covalently bound to PPi, is in direct proximity to plectasin, while GlcNAc is more distal to the complex interface. In accordance with these conclusions, plectasin can bind to the cognate cell wall precursor lipid I (Extended Data Fig. 4c,d), which lacks GlcNAc, although with reduced affinity^5^, suggesting a role of GlcNAc beyond the direct interaction with plectasin.

Next, we analysed the role of the lipid II pentapeptide. Therefore, we co-assembled ^13^C,^15^N-lipid II with NMR-inactive ^12^C-plectasin and acquired ssNMR spectra that exclusively reported on the pentapeptide in the complex (Fig. 2f, Extended Data Fig. 4b and Supplementary Fig. 1). Dipolar 2D CC spectra, which show only rigid residues, suggested that the first four pentapeptide residues are immobilized. This is a higher degree of immobilization than in the presence of teixobactin, which was shown not to interact with the pentapeptide^11,12^ (Fig. 2f and Extended Data Fig. 4b). The pentapeptide’s immobilization upon plectasin binding was confirmed in a complementary scalar NMR spectrum^17^ that solely reports on mobile residues and in which we only observed the C-terminal residues A4 and A5 and the side chain of K3. Our data hence show that the backbone of residues A1-K3 is rigid, A4 is in the intermediate regime, and A5 is flexible (Fig. 2g). Together, the global rigidity of the pentapeptide strongly suggests that it is directly involved in the plectasin–lipid II interface.

### Calcium ions enhance activity

**Fig. 3:**
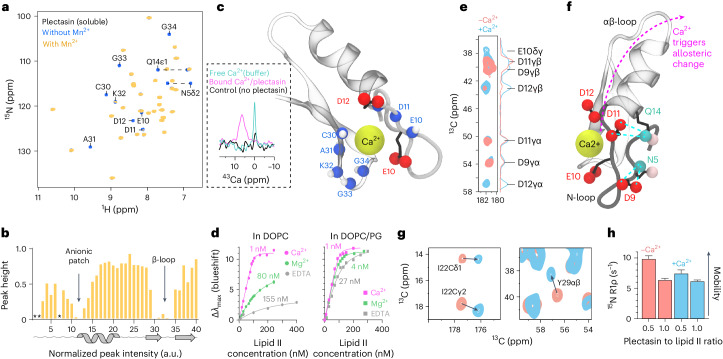
Ca^2+^ binds plectasin and increases lipid II affinity. **a**, 2D NH solution NMR spectra of unbound plectasin in the absence (blue) and the presence (yellow) of paramagnetic Mn^2+^. **b**, Signal intensities derived from the NMR spectrum in **a** in the presence of Mn^2+^ show that Mn^2+^ binds the anionic patch and the β-loop. Signals marked with asterisks (*) were not assigned. a.u., arbitrary unit. **c**, Illustration of the ion binding site. Ca^2+^ was manually modelled. The insert shows a ^43^Ca ssNMR spectrum of Ca^2+^ (in magenta) bound to the plectasin–lipid II complex in DOPG membranes; the control spectrum (in black) in DOPG membranes was recorded in the absence of plectasin; a spectrum of free Ca^2+^ in buffer is shown in cyan. **d**, Plectasin to lipid II binding affinities measured by tryptophan fluorescence (left) in zwitterionic DOPC membranes and (right) in anionic DOPC/DOPG membranes (1:1 mixture) with 1 mM Ca^2+^ (magenta), 1 mM Mg^2+^ (green) and in the absence of bivalent ions (100 μM EDTA, grey). Source data are provided. **e**, Dipolar ssNMR data show a rigidification of the anionic side chains E10 and D12 of lipid II-bound plectasin in the presence of Ca^2+^, confirming that bivalent ions bind to the complex. **f**, Side chains D9/D11 define the conformation of the N-loop, while E10/D12 bind to Ca^2+^. **g**, Zooms into 2D PARISxy^45^ CC ssNMR data show that Ca^2+^ binding allosterically modulates the αβ-loop (I22) and the β-sheet (Y29), in red without Ca^2^^+^ and in blue with Ca^2^^+^. **h**, ssNMR ^15^N *T*_1ρ_ dynamics show that Ca^2+^ rigidifies plectasin at sub-stoichiometric plectasin to lipid II concentrations. The error bars show the standard error of the fit. Source data are provided. Source data

**Extended Data Fig. 5:**
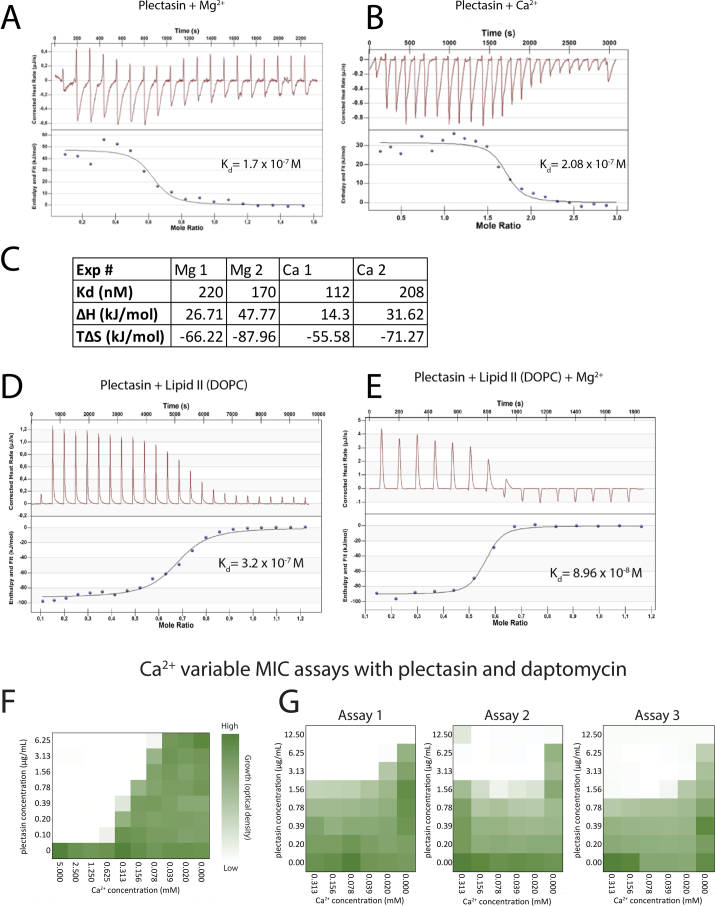
Plectasin binds to bivalent cations with high affinity and its activity is enhanced by calcium ions. Representative ITC traces of the binding of bivalent cations to plectasin in solution and the effect of Mg^2+^ on plectasin-lipid II binding. Heat releases and fits of Mg^2+^ (**A**) or Ca^2+^ (**B**) titrated to plectasin in solution. **C)** Calculated affinities and thermodynamic parameters experiments from (A) and (B), conducted in duplicate. Heat release traces and fits of the titration of Lipid II DOPC vesicles to plectasin without (**D**) and with (**E**) the addition of Mg^2+^. **F,G)**. Ca^2+^ variable assays with daptomycin and plectasin. **F)** The assay was established with daptomycin, whose antimicrobial activity strongly depends on the presence of Ca^2+^. We observe a marked ( > 60 fold) increase in antimicrobial activity with increasing Ca^2+^concentration, validating the assay. Note that daptomycin is strictly Ca^2+^-dependent, with an increase in MIC by >100 if Ca^2+^ is replaced by Mg^2+^ (ref. ^16^). Source data are provided. **G)** The assay (done in triplicate) demonstrates that plectasin’s antimicrobial activity improves with increasing Ca^2+^ concentration (4-8 fold improvement). Note that the effect of bivalent ions is presumably stronger, as the Ca^2+^ effect can be partially compensated by other bivalent ions such as Mg^2+^ (see Fig. 3D of the manuscript) that are required for bacterial growth. Source data are provided.

Plectasin features a long N-terminal loop, the function of which is unknown, but mutations in the N-loop decisively modulate plectasin’s activity^6–9^. The N-loop features a salient anionic patch (9-DEDD-12), of which we hypothesized that it could be a cation coordination site. First, we conducted ITC studies with plectasin in solution. Strikingly, ITC showed that plectasin binds bivalent cations Ca^2+^ and Mg^2+^ with high affinity (Kd = 159 ± 69 nM and 195 ± 35 nM, respectively) (Extended Data Fig. 5a–e). To identify the cation binding site, we performed solution NMR studies with paramagnetic Mn^2+^ ions that quench the signals of plectasin residues in proximity to the ion binding site (Fig. 3a,b). Upon addition of Mn^2+^, NMR signals of the anionic patch (E10, D11, D12) and the adjacent β-loop (30-CAKGG-34) disappeared, pinpointing the ion binding pocket right below the anionic patch (Fig. 3c). Finally, to directly detect Ca^2+^ binding, we acquired ^43^Ca ssNMR spectra in liposomes (with 4% lipid II). ^43^Ca ssNMR in membranes showed a major shift of the ^43^Ca signal in the presence of plectasin, conclusively demonstrating the formation of the plectasin–Ca^2+^–lipid II complex (Fig. 3c).

Next, we investigated whether Ca^2+^ and Mg^2+^, the most prominent bivalent ions in human serum, enhance the activity of plectasin. Therefore, we first investigated the binding affinity between plectasin and lipid II using intrinsic tryptophan fluorescence, a technique that, via a blueshift of the only tryptophan residue in plectasin (W8), reports on the peptide’s membrane insertion upon lipid II binding. Measurements were performed in neutral dioleoyl phosphatidylcholine (DOPC) liposomes and in anionic DOPC/dioleoyl phosphatidylglycerol (DOPG) liposomes. In neutral liposomes, we observed a major lipid II binding affinity increase by >100-fold in the presence of Ca^2+^ (Kd = 1 nM with Ca^2+^; 155 nM without bivalent ions). This affinity increase is highly specific for Ca^2+^, while Mg^2+^ had a much smaller effect (Kd = 80 nM). Strikingly, in anionic liposomes that mimic bacterial membranes better than neutral liposomes, the binding affinity improves (Kd = 27 nM without bivalent ions) and is less specific for the type of bivalent ion (Kd = 4 nM with Mg^2+^), while the highest affinity is still observed in the presence of Ca^2+^ (Kd = 1 nM).

Afterwards, we conducted minimum inhibitory concentration (MIC) studies in Ca^2+^-depleted media^18^ to investigate directly whether the increase in binding affinity is paralleled by enhanced antimicrobial activity in the presence of Ca^2+^. The functional assays were validated with the antibiotic daptomycin^19^, the activity of which strictly depends on the presence of Ca^2+^ (Extended Data Fig. 5f,g). We note that Ca^2+^-depleted media contain a high concentration (0.5 mM) of Mg^2+^, which is essential for bacterial growth. Although to a lesser extent than in the presence of Ca^2+^, plectasin’s lipid II affinity improves in the presence of Mg^2+^, which suggests that the total impact of bivalent ions is underestimated in Extended Data Table 1. This is presumably different for daptomycin^19^, which has a very high specificity for Ca^2+^ over Mg^2+^ (ref. ^19^).

To understand at the structural level how Ca^2+^ binding affects complex formation and activity, we acquired 2D CC ssNMR spectra. After adding Ca^2+^, the signals of plectasin’s anionic E10 and D12 side chains became more intense, implying rigidification by direct Ca^2+^ coordination and confirming the Ca^2+^-binding pocket (Fig. 3f). Conversely, the anionic side chains D9 and D11 on the opposite side of the binding pocket are already rigid in the absence of calcium due to electrostatic interactions with residues N5 and Q14 that define the conformation of the N-loop (Fig. 3e). Strikingly, Ca^2+^ binding also caused long-range allosteric structural changes in the β-sheet and in the αβ-loop that are both critically involved in lipid II binding (Fig. 3g). This establishes a clear link between Ca^2+^ coordination and target binding and offers an explanation how mutations in the N-loop, far away from the lipid II binding site, can increase the killing activity of plectasin^6–9^. In line with this, ssNMR data show that Ca^2+^ binding changes the dynamics of the entire plectasin molecule (Fig. 3h), causing a sizeable rigidification of the complex at a sub-stoichiometric (0.5:1) plectasin to lipid II ratio.

### Oligomerization upon target-binding

Recently, we showed that teixobactin and clovibactin form supramolecular fibrils to bind lipid II in membranes^11,13^. Given the global effects of Ca^2+^ on plectasin’s mobility and the sweeping signal differences compared to micellar data^5^ (Fig. 1c–e), we hypothesized that plectasin also uses a supramolecular mechanism.

**Fig. 4:**
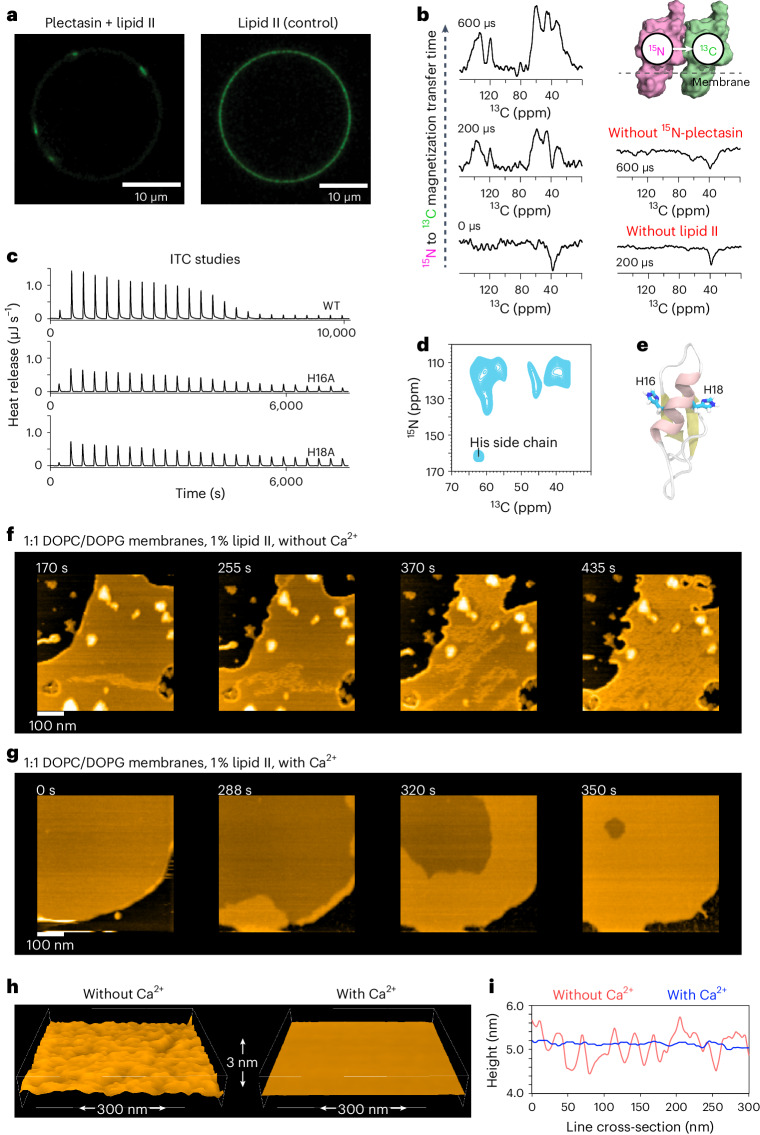
Plectasin oligomerizes in membranes upon lipid II binding. **a**, Confocal microscopy of GUVs doped with NBD-tagged lipid II shows that plectasin induces cluster formation. **b**, Plectasin oligomerization upon lipid II binding was probed in a Förster resonance energy transfer-like ssNMR set-up^21,22^ with an equimolar mixture of ^15^N- and ^13^C-labelled (‘mixed-labelled’) plectasin molecules. Left: a series of 1D DNP-ssNMR NHHC^21^ experiments with increasing ^1^H-^1^H magnetization transfer times applied to mixed-labelled plectasin in lipid II-doped liposomes show the presence of plectasin oligomers. Right (controls) The absence of signals without ^15^N-labelling or without lipid II show that this experiment specifically detects lipid II-bound oligomers. **c**, ITC data show that mutation of either H16 or H18 to an alanine strongly reduces lipid II binding affinity. **d**, A DNP-ssNMR 2D NHHC^21^ spectrum shows the involvement of histidine side chains in the oligomerization interface. His, histidine. **e**, The only histidine residues in plectasin, H16 and the conserved H18, are localized on opposite sides. **f**, Snapshots of a time-lapse HS-AFM video (Supplementary Video 4) following the oligomerization of plectasin in supported DOPC/DOPG lipid bilayers doped with 1% lipid II. The experiment was repeated three times (three independent samples; *n* = 3) with similar results. **g**, Same as **f** but in the presence of 1 mM Ca^2+^ (Supplementary Video 5). **h**, AFM height profiles show that plectasin forms a dynamic (Supplementary Video 6), loosely packed supra-structure (left) in the absence of Ca^2+^, while a uniform, densely packed supra-structure is formed in the presence of Ca^2+^. Images were taken from AFM measurements (**f** and **g**) after full coverage of the membranes by the plectasin supra-structures (Supplementary Video 7). The experiment was repeated three times (three independent samples; *n* = 3) with similar results. **i**, Cross-sections across images shown in **h** illustrate the markedly different behaviour of the supra-structures on the membrane surface with (in blue) and without (in red) Ca^2+^. Source data are provided. Source data

**Extended Data Fig. 6:**
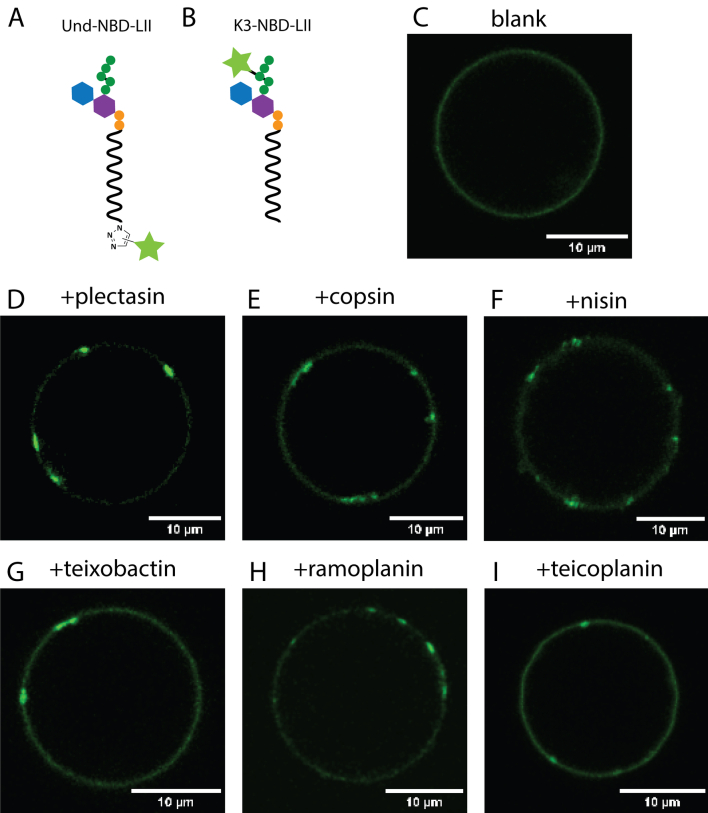
Fluorescence microscopy to probe oligomerization of lipid II binding drugs. GUVs consisting of 0.4 mol% Und-NBD-LII in DOPC were imaged after addition of the antibiotic. A representative blank is shown for untreated GUVs. **A**) Schematic structure of the lipid II dye used in this work with the tag at the end of the C55-isoprenyl tail (Und-NBD-LII) and **B**) of the previously reported lipid II dye with the NBD-tag attached to the sidechain of lysine-3 of lipid II (K3-NBD-LII) **C**) Blank **D**) 1 μM plectasin **E**) 1 μM copsin, a CSαβ-defensin also reported to bind to lipid II **F**) 1 μM nisin, a lantibiotic well-known to form supramolecular pores upon lipid II binding. **G**) 1 μM teixobactin, a depsi-peptide that targets lipid II and subsequently forms fibrils on top of the membrane. **H**) 1 μM ramoplanin, a glycolipodepsipeptide used in the clinic that binds lipid II. **I**) 1 μM teicoplanin, a glycopeptide also used in the clinic that targets lipid II.

To probe possible oligomerization of plectasin, we used giant unilamellar vesicles (GUVs) doped with nitrobenzoxadiazole (NBD)-tagged lipid II in combination with confocal microscopy. We first used a fluorescent lipid II variant with an NBD fluorophore attached to lysine-3 of the pentapeptide^11,12,20^. We did not observe any oligomerization with this set-up but surmised that tagging the pentapeptide, involved in the complex interface, could interfere with plectasin’s native mechanism. Therefore, we attached the NBD fluorophore at the end of the isoprenyl tail of lipid II (Extended Data Fig. 6a,b). Indeed, with this set-up, we observed cluster formation on the GUV surface after the addition of plectasin (Fig. 4a), suggesting a supramolecular mechanism.

We confirmed this supramolecular mechanism by ssNMR using a ^15^N(^1^H^1^H)^13^C (ref. ^21^) ssNMR experiment in combination with an equimolar mixture of ^13^C- and ^15^N-plectasin molecules added to lipid II-doped liposomes. Analogously to Förster resonance energy transfer, this experiment relies on intermolecular ^15^N to ^13^C magnetization transfer which can only occur if ^15^N-plectasin tightly binds ^13^C-plectasin (Fig. 4b)^22^. To avoid unspecific contacts due to crowding on the membrane surface, we worked with low lipid II concentrations (0.5 mol%). As low concentrations compromise NMR sensitivity, we compensated by dynamic nuclear polarization (DNP) enhancement^23,24^, which boosted ssNMR signal sensitivity by >50-fold (Supplementary Fig. 2). 1D NHHC DNP-ssNMR spectra show clear ^15^N to ^13^C magnetization transfer, confirming that plectasin oligomerizes upon lipid II binding, while control experiments showed no signals (Fig. 4b).

Next, we acquired a 2D NHHC DNP-ssNMR spectrum that shows that a histidine side chain is present at the plectasin–plectasin oligomerization interface (Fig. 4d and Supplementary Fig. 2). This is an important observation: plectasin is flanked by two histidine residues, H16 and the highly conserved H18 (ref. ^4^), which point in opposite directions (Fig. 4e). Substitution of either histidine by an alanine inactivates plectasin (Supplementary Table 1), suggesting crucial binding events on both flanks. This was confirmed by ITC that showed a >10-fold loss in lipid II binding affinity for the H16A and the H18A mutants (Fig. 4c). Given that both histidine residues, of which at least one is involved in oligomerization, critically reduce lipid II binding capacity, our data strongly suggest that efficient oligomerization and high lipid II binding affinity are interdependent, which is similar to teixobactin^11^.

It is noteworthy that previously it was suggested^5^ that H18 would be positively charged and thereby would form a salt bridge with the anionic γ-Glu2 of the lipid II pentapeptide. However, neither the protonation of H18 nor the salt bridge were experimentally observed. Indeed, our ssNMR data show that H18 and H16 side chains are neutral^25^ in the complex in membranes, refuting the purported H18–γ-Glu2 salt bridge (Supplementary Fig. 2).

### Oligomerization is Ca^2+^ sensitive and correlates to the MIC

**Extended Data Fig. 7:**
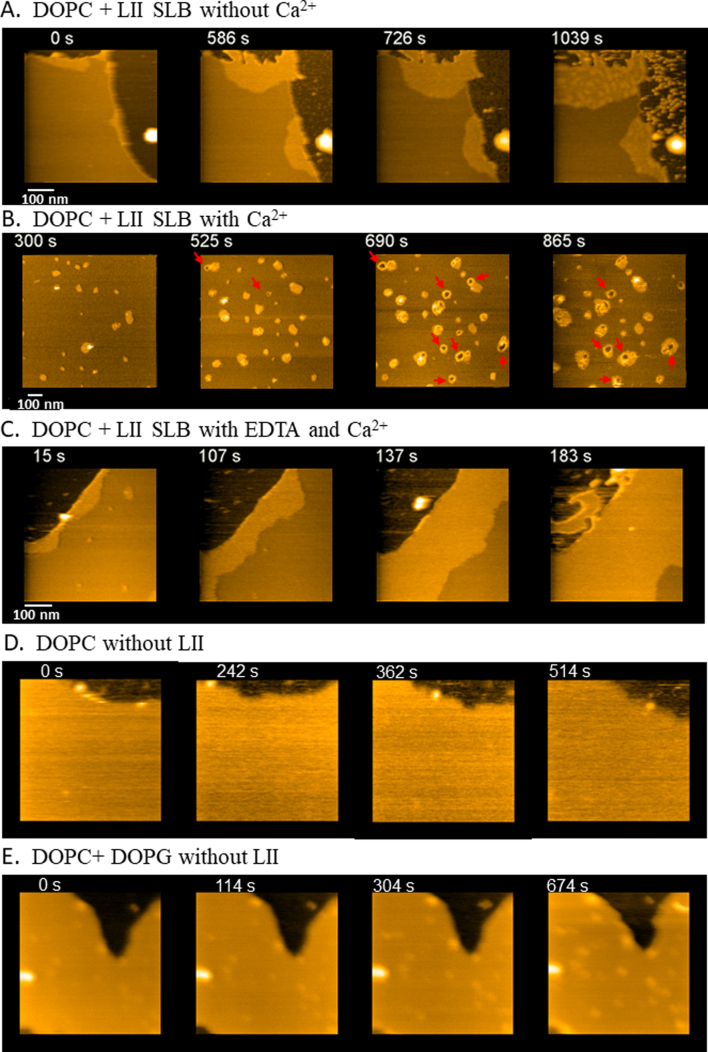
Ca^2+^ dependency of plectasin oligomerization on DOPC membrane containing 1% LII as captured by HS-AFM. **A)** snapshots of HS-AFM movies taken on supported lipid bilayer composed of DOPC with 1% LII after addition of 1 µM plectasin in absence of Ca^2+^. **B)** snapshots of HS-AFM movies taken on supported lipid bilayer composed of DOPC with 1% LII after addition of 1 µM plectasin in presence of 1 mM Ca^2+^. Red arrows indicate the created holes/pores after plectasin addition. **C)** snapshots of HS-AFM movies taken on supported lipid bilayer composed of DOPC with 1% LII after addition of 1 µM plectasin in presence of 1 mM Ca^2+^ and 10 mM EDTA (see also Supporting Video 3). **D)** snapshots of HS-AFM movies taken on supported lipid bilayer composed of DOPC without LII after addition of 1 µM plectasin. **E)** snap shots of HS-AFM movies taken on supported lipid bilayer composed of DOPC and DOPG (w/w 1:1) without LII after addition of 1 µM plectasin.

To better dissect the supramolecular mechanism, we used high-speed atomic force microscopy (HS-AFM), a dynamic technique that provides biomolecular-scale resolution in real time^26,27^, recently used to study the mechanism of teixobactin^11^, clovibactin^13^ and AMC-109 (ref. ^28^). Supported bilayers of zwitterionic DOPC lipids doped with 1% lipid II were incubated with plectasin and imaged. Within minutes, we observed the oligomerization of plectasin, starting from the membrane edge and then growing inwards into a sheet of approximately 2 nm thickness on top of the membrane (Extended Data Fig. 7a–c and Supplementary Video 1). Oligomerization was only observed in the presence of both plectasin and lipid II (see Extended Data Fig. 7d,e for control experiments). Next, we repeated the experiments using similar conditions (DOPC membranes with 1% lipid II) but with 1 mM Ca^2+^, where we observed pore formation (Extended Data Fig. 7a–c and Supplementary Video 2).

**Extended Data Fig. 8:**
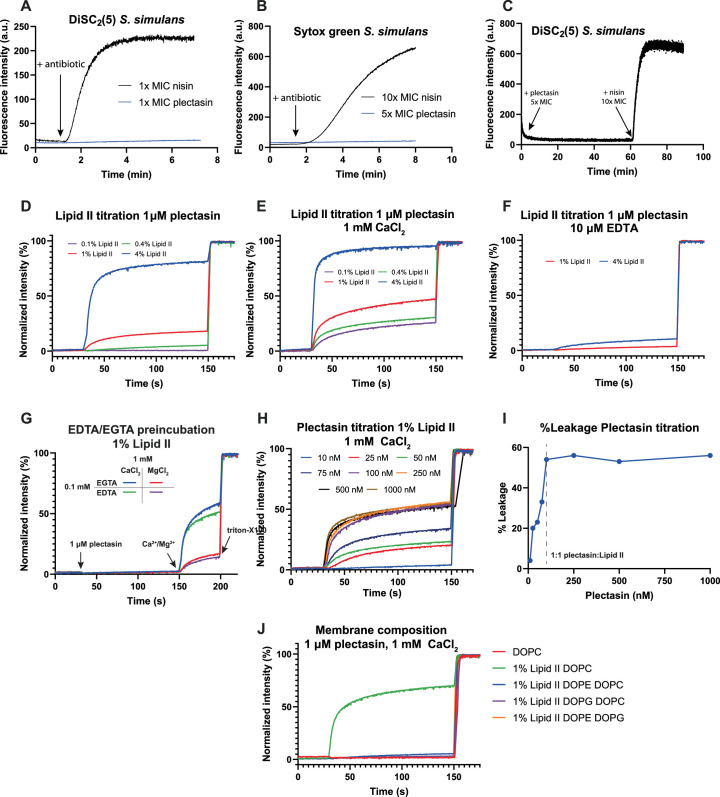
Carboxyfluorescein (CF) assays. **A,B,C)** Plectasin does not cause leakage in *S. simulans* membranes, even at significantly higher concentrations than the MIC (200 nM). Nisin is shown as positive control (MIC towards *S. simulans* is 75 nM). (**A**,**C**) The DiSC_2_(5) probe becomes fluorescent upon loss of membrane potential^22^. (**B**) SYTOX green becomes fluorescent upon binding to dsDNA^23^ inside bacteria. **D-J)** All assays were conducted in DOPC vesicles unless specified otherwise. Lipid II concentration is expressed as mol% of total lipid. Experimental details can be found in the methods section. Unless indicated otherwise plectasin was added at 30 seconds and 0.1 v% (final volume) Triton-X100 at 150 seconds. **D)** Titration of Lipid II concentration. **E)** same as D) but with Ca^2+^. **F)** same as A) but with EDTA. Increasing Lipid II concentration in the membrane promotes permeabilization by plectasin. At high calcium concentrations even membranes with low (and physiologically relevant) Lipid II concentrations are permeabilized. In contrast, in the absence of all calcium (with EDTA) modest leakage could be detected only at the highest Lipid II concentration. **G)** Vesicles pretreated with EDTA or EGTA were exposed to plectasin. After incubation for 120 seconds CaCl_2_ or MgCl_2_ was added. Bivalent cations are essential for plectasin-induced membrane permeabilization. Notably, MgCl_2_ induced leakage is starkly lower than for CaCl_2_, irrespective of the use of EGTA or EDTA, hinting at a differential effect of these bivalent cations on the mode of action of plectasin. **H)** Titration of plectasin to Lipid II vesicles. **i**, Percentage leakage as a function of plectasin concentration derived from the final leakage % of H). Leakage increases with plectasin concentration until all Lipid II on the outside of the vesicles is occupied (half of the total Lipid II in the sample, as depicted by the dashed line). Leakage thus depends on the formation of a 1:1 Lipid II-plectasin complex. **J)** The effect of the membrane composition on leakage. While DOPC Lipid II vesicles are efficiently permeabilized by plectasin in the presence of calcium, virtually no leakage is observed when phospholipids native to gram-positive bacteria are included in the membrane. Source data are provided for all graphs A-J). Source data

However, pore formation is very unlikely a physiologically relevant action of plectasin, because previous assays in bacteria showed no sign of membrane permeabilization^5^, something that we confirmed (Extended Data Fig. 8a–c). Given that AFM studies were hitherto performed with lipids carrying phosphatidylcholine headgroups that are not native to bacteria, we next wondered whether plectasin’s action could be sensitive to the membrane composition. An impact of the membrane composition on oligomerization would also align with our tryptophan fluorescence assays that show a marked impact of the membrane environment on plectasin’s lipid II binding affinity (Fig. 3d) and our conclusions, based on NMR structure–activity studies (Fig. 4b–e), that binding affinity and oligomerization capacity are interdependent.

Therefore, we next conducted HS-AFM studies in membranes containing anionic lipids (1:1 DOPC/DOPG; 1% lipid II) in which half of the lipids carried phosphatidylglycerol headgroups that are common to bacterial membranes (Fig. 4f–i and Supplementary Videos 4 and 5). Indeed, while plectasin also rapidly oligomerized under these conditions, membrane pores were not observed in the presence of 1 mM Ca^2+^, showing that plectasin’s action is sensitive to the membrane composition. Strikingly, HS-AFM data showed that Ca^2+^ drastically changed the packing of the supra-molecular assembly, which forms a densely packed carpet on the membrane surface in the presence of Ca^2+^ while the supra-structure is dynamic and heterogeneous in the absence of Ca^2+^ (Fig. 4h,i). These observations agree well with ssNMR data (Fig. 3h) that showed a global rigidification of plectasin in the presence of Ca^2+^. Note that we also confirmed plectasin’s sensitivity to the membrane composition and to the Ca^2+^ concentration using carboxyfluorescein leakage assays as a complementary method (Extended Data Fig. 8d–j).

Altogether, as bacterial membranes are anionic and the concentration of bivalent ions high in human blood (>0.5 mM for each Ca^2+^ and Mg^2+^), the homogeneous supra-molecular carpet mechanism that we observe in (1:1 DOPC/DOPG; 1% lipid II) with 1 mM Ca^2+^ is likely representative of plectasin’s physiological action.

**Fig. 5:**
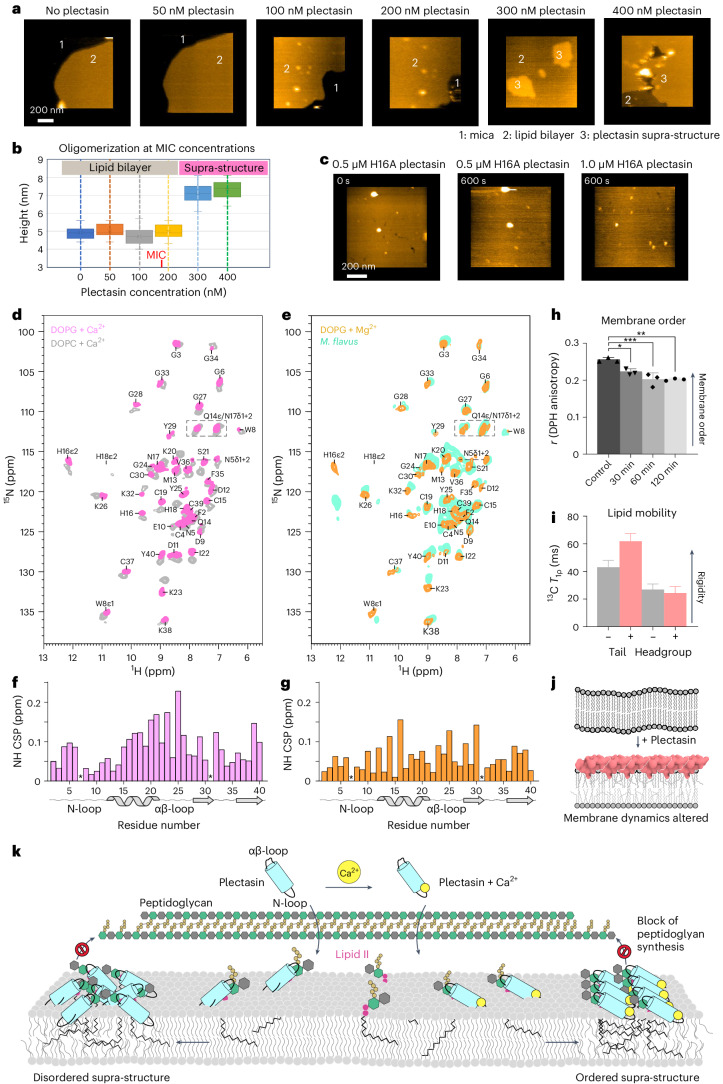
Cooperative oligomerization occurs around the MIC and affects the membrane state. **a**, Plectasin AFM titration: AFM images taken before (leftmost image) and 10 min after (other images) applying plectasin at varying concentrations across the MIC. Numbers indicate the regions mica (1), lipid bilayer (2) and plectasin supra-structure layer (3). The experiment was repeated three times (three independent samples; *n* = 3) with similar results. **b**, Height distribution of membrane surface and plectasin at varying concentrations show the sudden, cooperative emergence of oligomerization around MIC concentrations. The MIC of plectasin against *S. simulans* of 177 nM is indicated. The box plots extend from the 25th to the 75th percentiles, the whiskers indicate the minimum and maximum values, and the horizontal line indicates the median. The numbers of data points to generate the box plots are 46 (0 nM), 31 (50 nM), 20 (100 nM), 22 (200 nM), 16 (300 nM) and 30 (400 nM), respectively. Source data are provided. **c**, Snapshots of HS-AFM data taken before and 10 min after the addition of the H16A plectasin mutant at two different, high concentrations. No plectasin oligomerization is observed. The experiment was repeated three times (three independent samples; *n* = 3) with similar results. **d**, 2D NH ssNMR spectra of plectasin bound to lipid II in the presence of Ca^2+^ in anionic DOPG (magenta) or zwitterionic DOPC (grey) liposomes. **e**, 2D NH ssNMR spectra of plectasin bound to lipid II and the presence of Mg^2+^ in DOPG liposomes (orange) and in *M. flavus* membranes (light green). Note that the H16ε2 signal is similar in both spectra but shifted due to spectral backfolding. **f**, NMR signal changes (CSPs) of the two spectra shown in **d**. **g**, CSPs of the two spectra shown in **e**. **h**, DPH anisotropy membrane microfluidity measurements in *S. simulans* show changes in the membrane dynamics upon plectasin addition. Experiments were acquired in biological triplicates, depicted as mean ± s.d. Statistical analysis was performed using two-way ANOVA with Tukey’s multiple comparison testing, *P ≤ 0.05, **P ≤ 0.01, ***P ≤ 0.001 (30 min: *P* = 0.0199, 60 min: *P* = 0.0010, 120 min: *P* = 0.0011). Source data are provided. **i**, ssNMR dynamics data (^13^C-*T*_1ρ_) of ^13^C-phosphatidylglycerol lipids^52^ in liposomes (with 1% lipid II) in the presence and the absence of plectasin show that the formation of the plectasin–lipid II complex alters the lipid mobility. The error is the standard error of the fit. **j**, Scheme of the effect of plectasin binding to lipid II on membrane dynamics. Source data are provided. **k**, Model of the Ca^2+^-sensitive mode of action of plectasin. In the absence of Ca^2+^, plectasin binds to lipid II and then oligomerizes into a loosely packed supra-structure on the membrane surface. In the presence of Ca^2+^, plectasin oligomerizes into a densely packed carpet-like supra-structure that kills bacteria more effectively. Source data

Next, we probed whether oligomerization directly contributes to plectasin’s antimicrobial action. To this end, we measured AFM data as a function of the plectasin concentration using anionic DOPC/DOPG (1:1) membranes, doped with 1% lipid II, in the presence of 1 mM Ca^2+^ (Fig. 5a,b). Strikingly, we observed a sudden emergence of supra-structures above a plectasin concentration of 200 nM (partial coverage at 300 nM of plectasin concentration and a full coverage at 400 nM), a threshold that correlates well to the MIC of plectasin against *Staphylococcus* *simulans* that is 0.78 µg ml^−1^, corresponding to a plectasin concentration of 177 nM. However, oligomerization was not observed for plectasin mutant H16A, not even at a high concentration of 1 µM (Fig. 5c). Together, these data strongly suggest that the formation of supra-structures is an essential part of plectasin’s action. Furthermore, the sudden emergence of supra-structures above a certain threshold points to a cooperative oligomerization mechanism.

**Extended Data Fig. 9:**
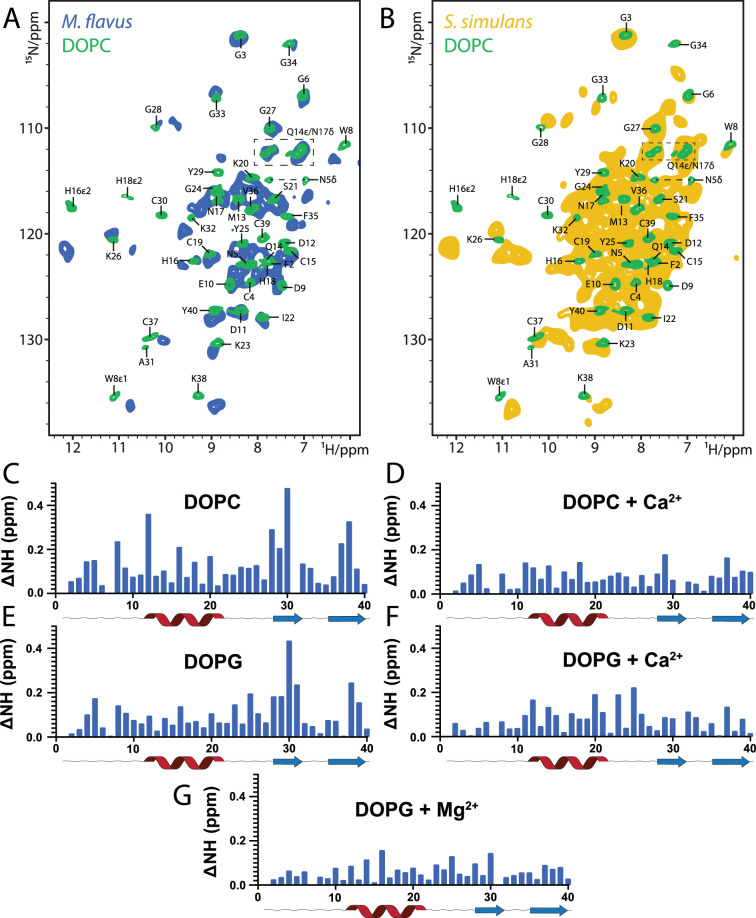
ssNMR of plectasin in bacterial membrane preparations. Comparison of ssNMR amide fingerprints of plectasin bound to Lipid II in bacterial membrane preparations and model membranes. Spectra of plectasin in bacterial membrane preparations were recorded at a magnetic field of 16.4 T (700 MHz ^1^H frequency), 60 kHz MAS and a sample temperature of 305 K. Spectra of plectasin in model membranes were recorded at 28.2 T (1200 MHz ^1^H frequency) 60 kHz MAS and a sample temperature of 305 K, with the exception of DOPG without calcium, which was recorded at 16.4 T (700 MHz ^1^H frequency). **A)** Overlay of an NH spectrum acquired in *M. flavus* membranes (blue) with an NH spectrum acquired in DOPC without added calcium (green) **B)** Same as **A**) but with spectrum acquired in *S. simulans* membranes (yellow). In *S. simulans* the spectrum is highly heterogeneous, while in *M. flavus* it is much more defined. Large apparent ^15^N chemical shift perturbations of the histidine sidechains in **A** and **B** are due to differences in spectral width of the indirectly detected dimension. **C)**, **D)**, **E), F) & G)** Calculated CSPs of plectasin in M. flavus membranes compared to in DOPC (**C**), DOPC with Ca^2+^ (**D**), DOPG (**E**), DOPG with Ca^2+^ (**F**), and DOPC with Mg^2+^ (**G**). Source data are provided for C-G). Source data

Afterwards, we investigated the impact of the membrane composition at high resolution by ssNMR and observed signal changes in the entire plectasin molecule in anionic compared to zwitterionic lipids (Fig. 5d,f and Supplementary Fig. 3), in line with a differential supramolecular packing in anionic membranes. The most pronounced signal changes were observed in plectasin’s αβ-loop that is involved in lipid II binding, linking the impact of the membrane composition on oligomerization with target binding. Next, we acquired ssNMR spectra of the plectasin–lipid II complex directly in native cell membranes (Fig. 5e,g and Extended Data Fig. 9)^12^ in the form of plasma membranes of *Micrococcus flavus* that are strongly anionic^29^. ssNMR signals in bacterial membranes overlapped closely with signals acquired in DOPG membranes in the presence of bivalent ions, showing that the ssNMR data in anionic membranes with Ca^2+^/Mg^2+^ are representative of the physiological action of plectasin. Furthermore, the high quality of the cellular ssNMR spectrum shows that the complex is well ordered in *M. flavus* membranes. Note that we acquired native cell membrane spectra with Mg^2+^ because Mg^2+^ is present in high concentrations during sample preparation.

We hypothesized that plectasin binding and the formation of dense antibiotic–lipid II supra-structures interferes with the natural organization of the plasma membrane. We conducted fluorescence anisotropy assays in bacteria using DPH (1,6-diphenyl-hexa-1,3,5-triene), a compound that reports on membrane fluidity. Addition of plectasin to intact *S. simulans* cells resulted in a decrease in the fluorescence anisotropy, implying that plectasin affects the overall membrane fluidity (Fig. 5h). The change in membrane fluidity was confirmed by complementary ssNMR longitudinal relaxation in the rotating frame (*T*_1ρ_) relaxation measurements of phosphatidylglycerol lipids in liposomes (1% lipid II), in which we observed clear changes in the dynamics of the lipid tail and headgroup dynamics upon plectasin binding (Fig. 5i). Together, our data show that plectasin’s supramolecular action upon lipid II binding affects the state of the membrane (Fig. 5j).

### A widespread mechanism

Our study establishes that plectasin uses a supramolecular action. Supramolecular mechanisms are known for a few lipid II binding lantibiotics such as nisin^30^ and were recently discovered for the depsipeptides teixobactin^11,12^ and clovibactin^13^. We hence wondered whether oligomerization could be a more general mechanism to sequester lipid II. We used fluorescence microscopy to show oligomerization for different lipid II binders including the αβ-defensin copsin^31^ and the glycopeptides teicoplanin and ramoplanin^32^ (Extended Data Fig. 6c–i). All these lipid II binders, of different (bacterial, fungal and semi-synthetic) origins and chemical structures, caused the formation of lipid II domains in liposomes, suggesting oligomerization as a prevalent mechanism to capture cell wall precursors.

## Discussion

The mechanistic data revealed in this study illuminate how plectasin targets the indispensable cell wall precursor lipid II in membranes and paints the following picture (Fig. 5k). Settling on the membrane surface, plectasin uses long hydrophobic residues in the N-loop and αβ-loop to anchor into the membrane. Then, plectasin directly binds to conserved pyrophosphate, the MurNAc sugar and the pentapeptide of lipid II. ssNMR, ITC, fluorescence spectroscopy and mutagenesis data show that the long αβ-loop of plectasin adopts a well-defined conformation that is critically required for lipid II binding, probably by stabilizing interactions between cationic lysine side chains and the anionic PPi or the γ-glutamate of the lipid II pentapeptide. The specific binding of Ca^2+^ ions to the anionic patch (9-DEDD-12) in plectasin’s N-loop drastically increases plectasin’s binding affinity and activity. Ca^2+^ binding to monomeric plectasin rigidifies the N-loop and displaces water molecules, presumably reducing the entropic penalty for the formation of the oligomeric supra-structure. Allosterically, Ca^2+^ binding to the N-loop also changes the conformation of plectasin’s β-sheet and αβ-loop, stabilizing the interaction with lipid II. Thereby, our data establish the molecular underpinning why mutations in the N-loop, far away from the lipid II binding site, improve plectasin’s activity^6–9^. In addition, HS-AFM and ssNMR show that high-affinity binding of Ca^2+^ to plectasin fosters the formation of dense carpet-like plectasin–lipid II supra-structures on the membrane surface. Plectasin oligomerization emerges at concentrations close to the MIC, strongly suggesting that it is an essential part of plectasin’s antimicrobial action. Oligomers emerge above a sharp concentration threshold, hinting at a cooperative event. The formation of rigid supra-structures is presumably irreversible on biological timescales, acting as a sink that concentrates lipid II, a mesh in which lipid II gets irretrievably entangled. These supra-structures interfere with the natural fluidity of the bacterial plasma membrane which presumably contributes to the action of plectasin^33^. Oligomerization critically requires the presence of conserved histidine residues that flank plectasin on opposition sides. Replacing the histidine residues with alanines markedly reduces lipid II binding affinity, implying that lipid II binding affinity and oligomerization capacity are interdependent, comparable to teixobactin, the activity of which also depends on its oligomerization capacity^11,34^. Plectasin would hence not bind to soluble PPi or PPi-containing nucleosides that are commonly present in the environment. Instead, the tight binding of plectasin to the PPi group requires the efficient formation of a supramolecular structure on the membrane surface, substantially decreasing the dissociation rate constant *k*_off_, that is, increasing the residence time (1/*k*_off_) of plectasin at its target side. Thereby, our comprehensive molecular analysis elucidates the fascinating question of how plectasin manages to tightly and selectively bind the sugar-PPi moieties of cell wall precursors (lipid II and lipid I) and the lack of plectasin toxicity against mammalian cells^4^. Plectasin’s action against lipid II expands our knowledge of cell-wall-targeting antibiotics and points the way to the design of effective new antimicrobial compounds with high selectivity. As we demonstrate with a broad array of lipid II-binding antibiotics, the formation of supra-structures emerges as a widespread mechanism to effectively and safely target cell wall precursors.

A shortcoming of our study is the limited high-resolution knowledge on the different interfaces that form the plectasin–lipid II–Ca^2+^ supra-structure. Currently, we ignore the structural details how bivalent ions modulate the supra-structure; for example, we do not know whether bivalent ions form some kind of bridge between plectasin molecules. We are also uncertain about the finer structural details of the plectasin–plectasin interaction and the plectasin–lipid II interaction. Furthermore, while it is clear from our data that oligomerization is an indispensable part of plectasin’s action, it is uncertain whether the modulation of the membrane properties by the supra-structure contributes to the antimicrobial action.

Our study also shows that plectasin can follow different oligomerization pathways, depending on the experimental conditions, that is, depending on the membrane environment and depending on the presence or the absence of bivalent cations. We emphasize that this does not mean that plectasin would deploy different oligomerization pathways under physiological conditions, as plectasin’s physiological action will always unfold in the presence of bivalent ions and in bacterial membranes that are usually anionic. However, it is a clear call to perform mode of action studies of drugs that act on membranes using experimental set-ups that closely resemble native conditions.

## Methods

### Plasmid construction

The coding sequence of plectasin was obtained by gene splicing by overlap extension^35^ and polymerase chain reaction gene synthesis. For the latter, Klenow fragment is used to synthesize the double-stranded DNA. Two primers were designed to amplify the plectasin gene. Afterwards, polymerase chain reaction was performed with the fully synthesized gene sequence for the addition of 3′ adenine overhangs by Taq DNA polymerase (ThermoFisher). Plectasin was cloned into the Champion pET SUMO TA expression vector by TA overhang cloning according to the manufacturer’s specifications. Mutagenesis of plectasin was performed as described in the QuikChange II Site-Directed Mutagenesis Kit (Agilent Technologies) manual using KOD Hot Start DNA Polymerase (Novagen). Reaction mixtures were thermocycled and subsequently digested with type II methyl-directed restriction enzyme DpnI for 1 h at 37 °C. *E. coli* DH5α cells were transformed, positive colonies were selected and plasmid DNA was isolated. All plasmids were sequenced to ensure the coding sequence of plectasin mutants were correct and in-frame.

### Plectasin preparation and purification

PET SUMO–plectasin was transformed into *E. coli* SHuffle T7 cells (NEB) by heat shock according to the manufacturer’s specifications. Overexpression of the 6xHis-SUMO-plectasin fusion protein was accomplished following the protocol of ref. ^36^. Expression of 6xHis-SUMO-plectasin was induced in 1 l ^13^C,^15^N-labelled M9 medium at an OD_600_ of 0.6 using 0.5 mM isopropyl thiogalactoside for a period of 4 h at 37 °C. Cells were collected by centrifugation at 4,000 × *g* at 4 °C for 30 min. Bacterial pellets were resuspended in lysis buffer (50 mM sodium phosphate, 150 mM NaCl, 25 mM imidazole and 1 mM β-mercaptoethanol, pH 8.0) supplemented with 200 μg ml^−1^ chicken egg white lysozyme and 1 μg ml^−1^ DNase I. The suspension was sonicated, and cellular debris was removed by centrifuging at 40,000 × *g* for 30 min. The supernatant was filtered through a 0.45 μm filter. The 6xHis-SUMO-plectasin fusion protein was purified by affinity chromatography using pre-equilibrated Ni-NTA resin (Qiagen). The sample was repeatedly (five times) applied to the resin and subsequently washed with 20 column volumes of wash buffer (50 mM sodium phosphate, 150 mM NaCl, 25 mM imidazole and 1 mM β-mercaptoethanol, pH 8.0) and eluted with 2 column volumes of elution buffer (50 mM phosphate buffer, 150 mM NaCl, 400 mM imidazole and 1 mM β-mercaptoethanol, pH 8.0). Afterwards, SUMO protease was added at a 1:1,000 molar ratio to the purified fusion protein and incubated overnight at 4 °C. Further purification of plectasin was accomplished by size exclusion chromatography on a Superdex 30 Hiload 26/60 column at a flow rate of 2.6 ml min^−1^ with 50 mM sodium phosphate, 150 ml NaCl and 1 mM β-mercaptoethanol, pH 7.2. Fractions corresponding with pure plectasin were collected and stored at −20 °C and concentrated and/or buffer exchanged to the desired concentration using a 3.5 kDa cut-off Amicon Ultra filter unit (Millipore) before use.

### Synthesis of lipid II and cell wall precursors

Lipid II was synthesized and purified as previously described^37^. In short, lipid II was reconstituted enzymatically from undecaprenyl phosphate, uridine diphosphate (UDP)-GlcNAc and UDP-MurNAc-pentapeptide with the N-acetylglucosamine transferase MurG and the phospho-N-acetylmuramoyl-pentapeptide-transferase mraY and purified on diethylaminoethyl cellulose. Undecaprenyl was isolated from *Laurus nobilis* and subsequently enzymatically phosphorylated^38^. ^13^C^15^N-labelled UDP-GlcNAc and UDP-MurNAc-pentapeptide were extracted from *Bacillus* *cereus* and *S. simulans*, respectively, and grown in ^13^C^15^N-labelled medium consisting of M9 supplemented with ^13^C^15^N-Silantes, ^13^C^15^N-Bioexpress, U-^13^C-glucose and ^15^N-NH_4_Cl after accumulation of the precursors using fosfomycin or vancomycin and moenomycin. Undecaprenol ω-azide was synthesized as previously described^39^. Trichloroacetonitrile (784 µl, 78.0 μmol, 6.0 equivalent) was added to a solution of undecaprenol ω-azide (10.0 mg, 13.0 μmol) in anhydrous dichloromethane (1 ml), and the resulting solution was stirred at ambient temperature for 1 h. A solution of tetrabutylammonium dihydrogen phosphate (17.7 mg, 52.0 μmol, 4.0 equivalent) in anhydrous dichloromethane (1 ml) was then added dropwise, and the resulting solution was refluxed overnight. The reaction mixture was concentrated in vacuo, redissolved in tetrahydrofuran (1 ml) and 25% aqueous ammonium hydroxide (0.2 ml) and stirred for 30 min. Toluene (2.5 ml) and methanol (2.5 ml) were then added, and the mixture was stirred for 20 min, filtered and concentrated in vacuo. The resulting oil was washed with petroleum ether (3 × 5 ml) and redissolved in methanol (2 ml). Excess ammonium form DOWEX 50WX8 resin was added, and the mixture was stirred for 30 min, filtered and concentrated in vacuo. The resulting crude oil was purified by flash column chromatography (SiO_2_, 65:25:5 chloroform/methanol/0.1% aqueous ammonium hydroxide) to yield the product as a clear oil (5.0 mg, 44%). Und-NBD-lipid II was synthesized by reconstitution from UndP-N_3_ as described for native lipid II, followed by addition of the tag via copper-catalysed cycloaddition with alkyne-NBD. N_3_-LII, CuSO_4_ and tris-hydroxypropyltriazolylmethylamine were premixed in 10 mM Tris–HCl buffer at pH = 8.0 and stirred at room temperature for 20 min. Then, sodium ascorbate was added, and the mixture was dried under a flow of N_2_. The residue was redissolved in H_2_O and added 1:1 (*v*/*v*) to a premixed solution of Und-N_3_-lipid II and NBD-alkyne in MeOH to final concentrations of CuSO_4_ (0.1 mM), tris-hydroxypropyltriazolylmethylamine THPTA (0.5 mM), sodium ascorbate (1 mM), Und-N_3_-lipid II (0.1 mM) and NBD-alkyne (0.3 mM). The reaction was protected from light, stirred at room temperature overnight and purified as described for unmodified lipid II. Lipid concentrations were measured as inorganic phosphate after treatment with perchloric acid^40^.

### Isothermal titration calorimetry

About 200 nm large unilamellar vesicles (LUVs) were prepared from 2 mol% lipid II in DOPC by the extrusion technique^41^. ITC measurements were conducted on a low-volume Affinity ITC (TA instruments). All measurements were conducted at 37 °C. Samples were degassed for 10 min before the ITC experiments. The cell was filled with plectasin (in 50 mM HEPES, 150 mM NaCl at pH = 7.2) and titrated with LUVs in the same buffer under constant stirring at 125 r.p.m. The data were analysed using the Nano Analyze software version 3.11.0 (TA Instruments). After baseline correction, the integrated peaks were fitted using an independent binding model. Each measurement was acquired in duplicate.

### NMR sample preparation

Solution NMR samples were prepared by adding 10% D_2_O to 20–50 µM plectasin in 50 mM phosphate, 150 mM NaCl at pH = 7.2.

Unless specified otherwise, ssNMR samples contained multi-lamellar vesicles (MLVs) of DOPC doped with 4 mol% lipid II (lysine form) at a final molar ratio of 1:1 plectasin/lipid II in phosphate buffer (50 mM sodium phosphate, 150 mM NaCl at pH = 7.2). For experiments done in the presence of Ca^2+^, we used HEPES buffer (50 mM HEPES, 150 mM NaCl at pH = 7.2). Appropriate lipid stocks in chloroform or 2:1 chloroform/methanol were mixed in a glass tube, dried under a flow of N_2_ with gentle heating and then exposed to high vacuum for 20 min to form lipid films. Films were then hydrated by the addition of plectasin in buffer, followed by extensive vortexing. The resulting vesicles were collected by ultracentrifugation at 100,000 × *g* for 30 min at 4 °C. This co-assembly sample preparation strategy ensures that plectasin is present on both sides of the membranes, including in the inner vesicles of MLVs, and thus is able to reach all the lipid II molecules in the MLVs. Subsequently, liposomes were directly centrifuged into ssNMR magic angle spinning (MAS) rotors. For the ^15^N(^1^H^1^H)^13^C experiments, we used 0.5 mol% lipid II in DOPC and an equimolar mixture of ^13^C-enriched plectasin (^14^N^13^C-plectasin, that is, N at natural abundance) and ^15^N-enriched plectasin (^15^N^12^C-plectasin, that is, C at natural abundance) in the presence of 15 mM AMUPol^42^ in 60% glycerol-d8, 35% buffer in D_2_O (to the same final salt concentrations described above) and 5% H_2_O. Samples were loaded into sapphire 3.2 mm rotors. For ^43^Ca ssNMR experiments, 0.5 mM ^43^CaCO_3_ (84.8 ± 0.8% isotopic enrichment) was added to plectasin in buffer before hydration of 4 mol% lipid II in DOPG lipid films. As a control, we made an identical sample only omitting plectasin in the buffer. After sample collection by centrifugation, only plectasin-bound calcium is concentrated in the pellet. In addition, one sample was made containing only 10 mM ^43^Ca in buffer as a control for unbound Ca^2+^.

Bacterial membrane vesicles with reconstituted lipid II were prepared essentially as previously described^29^. In short, *M. flavus* DSM 1790 or *S. simulans* 22 were grown to late log phase in tryptic soy broth (TSB), collected by centrifugation, washed once with buffer (50 mM phosphate, 50 mM NaCl pH 8.0) and lysed using a cell disruptor (Constant Systems) in the presence of lysozyme, benzonase and 1 mM MgSO_4_. Debris was removed by low-speed centrifugation (4,000 × *g*, 4 °C, 15 min), and large membrane vesicles were collected by high-speed centrifugation (40,000 × *g*, 4 °C, 1 h). Lipid II was reconstituted in the presence of 3 mM MgSO_4_ and an excess of lipid II precursors UDP-MurNAc-pentapeptide and UDP-GlcNAc. Reagents were mixed with four freeze–thaw cycles on ice, followed by a 30 min incubation at room temperature. ^15^N-plectasin was then added, and after two more freeze–thaw cycles, the vesicles were collected by ultracentrifugation (175,000 × *g*, 4 °C, 90 min) and spun down into 1.3 mm rotors.

### Solid state NMR experiments

^1^H-detected experiments were carried out at 60 kHz MAS, magnetic fields of 16.4, 18.8 and 28.2 T (700, 800 and 1,200 MHz ^1^H frequency) and a sample temperature of approximately 305 K. Phase-inverted supercycled sequence for attenuation of rotary resonance low-power proton decoupling was applied in all dimensions^43,44^. ^13^C-detected experiments were carried out at 16.4, 18.8 or 22.3 T (700, 800, or 950 MHz ^1^H frequency). Dipolar-based 2D ^13^C-^13^C spectra were recorded at a sample temperature of 270 K using phase-alternated recoupling irradiation scheme using orthogonal radio-frequency phases (PARISxy)^45,46^ recoupling (*m* = 1 at 16.4 T and *m* = 2, 10 kHz recoupling amplitude, 20–1,000 ms mixing time) and small phase incremental alternation with 64 steps decoupling^47^. Chemical shift assignments were obtained using ^1^H-detected 3D CαNH, Cα(CO)NH and CO(Cα)NH experiments as previously described^48^, combined with 2D ^13^C-^13^C PARISxy^45,46^ experiments with different mixing times. A 2D scalar ^13^C^13^C total through-bond-correlation spectroscopy (TOBSY)^17^ experiment was conducted with 8 kHz MAS at 295 K sample temperature with 6 ms ^13^C-^13^C mixing time. Mobility-edited 2D ^1^H(^1^H)^13^C experiments^49^ were performed at 700 MHz, 16.5 kHz MAS and a sample temperature of 300 K. A ^1^H *T*_2_ relaxation filter of 3.5 ms was used. Magnetization from mobile lipids and water was transferred to the rigid antibiotic with a ^1^H-^1^H mixing time of 7 ms, followed by a short cross-polarization step (175 μs) to ^13^C nuclei. DNP-enhanced ^15^N(^1^H^1^H)^13^C experiments^21^ were carried out at 9.4 T (400 MHz ^1^H frequency) at 8 kHz MAS and at 100 K. Very short contact times (80 μs) for the second and third cross polarization (CP) steps were used, and the ^1^H-^1^H mixing time was set to 200 μs. 1D ^43^Ca ssNMR spectra were recorded in 2.5 mm rotors, at 18.8 T (800 MHz ^1^H frequency), 10/15 kHz MAS and a sample temperature of 285 K using direct polarization and no decoupling. ^31^P ssNMR spectra were recorded at 11.7 T for 1D CP spectra or 18.8 T for REDOR experiments (500 or 800 MHz ^1^H frequency, respectively). 1D CP ^31^P spectra were recorded with 13.5 kHz MAS and a sample temperature of 280 K. ^31^P{^13^C} REDOR^50^ experiments were conducted at 60 kHz MAS and a sample temperature of 290 K using 15 kHz swept-frequency two-pulse phase modulation^51^ ^1^H decoupling during dephasing and acquisition. Dephased (*S*_r_) and reference (*S*_0_) spectra were recorded using 0, 4, 8, 12, 16 and 20 ms dephasing times. The analysis of the REDOR data is described in Extended Data Fig. 3d. ^1^H-detected ^15^N *T*_1ρ_ relaxation experiments^15^ were used to measure the dynamics of lipid II-bound plectasin in membranes at fast (60 kHz) MAS. ^1^H-detected ^13^C *T*_1_ and *T*_1ρ_ experiments were used to measure the dynamics of ^13^C-labelled phosphatidylglycerol lipids^52^ in liposomes (89 mol% DOPG, 10 mol% ^13^C-labelled phosphatidylglycerol isolated from *S. simulans*, 1 mol% lipid II) with and without plectasin. Spectra were recorded at 16.4 T (700 MHz ^1^H frequency), 58 kHz MAS and a sample temperature of approximately 305 K. Relaxation data were fitted in Prism version 9.5.1, and the error shows the standard error of the fit.

### Solution state NMR experiments

Backbone assignments of plectasin were obtained using 3D CαNH, CαCβNH, CONH and CβCα(CO)NH experiments at magnetic fields of 21.1 or 14.1 T (900 or 600 MHz ^1^H frequency). Backbone amide dynamics were determined at 14.1 T (600 MHz ^1^H frequency) at 298 K. ^15^N *T*_1_ relaxation times were determined using relaxation delays of 0.01, 0.05 (×2), 0.10, 0.20, 0.40 (×2), 0.7, 1.0 and 1.4 s. ^15^N *T*_2_ relaxation times were extracted from ^15^N *T*_1ρ_ measurements as previously described^53^. ^15^N *T*_1ρ_ relaxation times were determined using a spin-lock pulse of 1.9 kHz of 4, 16 (×2), 32, 48, 80, 112 (×2), 128, 160, 208 and 256 ms. Trajectories were fit to single exponentials.

### Microscopy

GUVs were prepared by electroformation using in-house build Teflon cells with two titanium wires as electrodes. About 1 μl of 0.5 mM DOPC with 0.4 mol% NBD–lipid II in 2:1 CHCl_3_/MeOH was brushed on both electrodes and thoroughly dried under vacuum. Next, the cell was filled with 350 μl 300 mM sucrose, and GUVs were formed by applying an oscillating voltage (sine wave, 2 V, 10 Hz) for 90 min and released from the electrodes (square wave, 2 V, 2 Hz). Microscopy slides were precoated using a 1 mg ml^−1^ BSA solution. Slides were filled with 300 μl 300 mM glucose solution, followed by addition of the antibiotic and finally 30–50 μl GUV solution. The GUVs were imaged using a Zeiss LSM 880 confocal microscope with a ×63/1.2NA glycerol objective lens. Green fluorescence (530–545 nm) was detected after excitation with a 488 nm laser.

### Carboxyfluorescein leakage

Vesicle leakage assays were conducted using LUVs filled with carboxyfluorescein. Carboxyfluorescein solutions were made by suspending ~50 mM carboxyfluorescein in H_2_O and adjusting the pH to ~7.2 using 10 M NaOH. The resulting solution was diluted with H_2_O to match the osmolarity of the outside buffer, which consisted of 10 mM Tris and 100 mM NaCl, at pH = 7.2. LUVs were prepared as described above. Non-enclosed carboxyfluorescein was removed using a Sephadex G50 column equilibrated in outside buffer. Measurements were conducted on a Cary Eclipse spectrophotometer at 20 °C under constant stirring by monitoring the fluorescence intensity at 515 nm (excitation at 492 nm, 5 nm slit width, 0.1 s averaging time). A total lipid concentration of 25 µM was used for all experiments. Fluorescence traces were normalized using the fluorescence baseline at the start as 0% and fluorescence after treatment with 0.1 vol% Triton-X100 as 100%.

### Tryptophan fluorescence spectroscopy

Plectasin’s single tryptophan can be used to monitor insertion into the membrane. Measurements were done on a Cary Eclipse (FL0904M005) fluorimeter at 20 °C under constant stirring in a 4 × 10 mm quartz cuvette. For titration experiments, the cuvette was oriented perpendicular to the excitation beam to minimize scattering. LUVs with or without 2 mol% lipid II (prepared as described above at 1–5 mM total lipid concentration) were titrated to 100 nM plectasin in 50 mM HEPES, 150 mM NaCl at pH = 7.2. Fluorescence was excited at 280 nm (5 nm slit) and recorded between 300 and 400 nm (10 nm slit) at 1 nm intervals with 1 s averaging per point. Spectra were corrected for a blank using buffer and for scattering using a titration series in the absence of plectasin. Corrected spectra were fit with log-normal distributions, and the shift of the resulting wavelength of maximum fluorescence (Δ*λ*_max_) as a function of lipid II concentration on the outside of the vesicles was fit using a quadratic binding equation^54^ to obtain the binding affinities.

### Bacterial permeabilization assay

Overnight cultures (37 °C, TSB) of *S. simulans* were diluted to OD_600_ = 0.1 and grown until mid-log phase (OD_600_ = 0.5–0.8) and collected by centrifugation (2,000 × *g*, room temperature, 5 min). Cell pellets were washed with buffer (10 mM Tris, 50 mM NaCl and 0.5% w/v glucose at pH = 7.2), resuspended to OD_600_ = 10 and stored at 4 °C until use. All assays were performed on a Cary Eclipse (FL0904M005) fluorometer. Measurements were done using bacterial suspensions at OD_600_ = 0.05 in the aforementioned buffer under constant stirring at 20 °C in a 4 × 10 mm quartz cuvette. About 0.2 µM 3,3′-diethylthiadicarbocyanine iodide or 0.25 µM SYTOX green was added and equilibrated for 2 min before addition of an antibiotic. For 3,3′-diethylthiadicarbocyanine iodide, we used excitation and emission wavelengths of 650 nm and 670 nm, respectively, while for SYTOX green, we used 504 nm and 523 nm. About 5 nm slits were used, and fluorescence signal was averaged every 0.3 s.

### DPH anisotropy measurements

Preparation of *S. simulans* stock solutions was done as described above for bacterial permeabilization in PBS. About 1 ml of a cell suspension of OD_600_ = 0.1 in PBS was treated with 1 µM plectasin and incubated at 37 °C. All assays were performed on a Cary Eclipse (FL0904M005) fluorometer fitted with a manual polarizer accessory (Varian). Measurements were conducted at 37 °C under constant stirring in a 4 × 10 mm quartz cuvette. About 10 µM DPH was added from a 1 mM stock in dimethyl sulfoxide (bringing the final dimethyl sulfoxide concentration to 1 vol%) and incubated for 5 min. Fluorescence was excited with 380 nm light (10 nm slit) and recorded between 430 and 520 nm (10 nm slit) with 0.5 s averaging time and data interval of 1.66 nm per point. Fluorescence anisotropy was calculated using the maximum intensity of each spectrum according to the following equation (where *I*_VV_ and *I*_VH_ represent the fluorescence intensity using parallel and orthogonal excitation and emission polarization filters, respectively):

$$r=\frac{{I}_{\rm{VV}}-{I}_{\rm{VH}}}{{I}_{\rm{VV}}+2{I}_{\rm{VH}}}$$

### MIC assays under standard conditions

Plectasin and analogues were serially diluted in cation-adjusted Mueller–Hinton broth (MHB) in round-bottom polypropylene 96-well plates (50 µl per well). Selected bacterial strains were grown from glycerol stocks on blood agar plates. Individual colonies were inoculated in TSB and incubated at 37 °C while shaking until OD_600_ reached 0.5. Bacterial suspensions were then diluted 100 times in cation-adjusted MHB and distributed (50 μl per well) over the microplate containing the diluted plectasin solutions. Plates were covered by a gas-permeable adhesive membrane and incubated at 37 °C while shaking. MICs were determined by visual inspection after 16–20 h (*Staphylococcus*) or 20–24 h (*Streptococcus*). For *Streptococcus* strains, the broth was additionally supplemented with 5% lysed horse blood, and the microplates were incubated under 5% CO_2_ atmosphere. In the assays, we used the following strains: *S. simulans*, *S. pneumoniae* serotype 8, *S. pneumoniae* ATCC6305 and *Streptococcus pyogenes* M3.

### Ca^2+^ variable MIC assays

Cation-depleted MHB was prepared as previously described^18^ and supplemented with MgCl_2_ (0.5 mM) and ZnSO_4_ (10 µM). Plectasin was serially diluted twofold over a 96-well polypropylene plate. Serial dilutions of Ca^2+^ in calcium-depleted medium were subsequently added to the wells containing the plectasin dilution series, followed by addition of bacterial suspensions. MICs were determined by visual inspection after 16–20 h (*Staphylococcus*). The effect of reduced Ca^2+^ levels on *S. simulans* growth in the absence of plectasin was assessed by monitoring growth at 37 °C by OD_600_ over 18 h (Supplementary Fig. 4).

### HS-AFM

HS-AFM imaging was performed on a supported lipid bilayer deposited on mica using an amplitude modulation tapping mode HS-AFM from RIBM (Ando type)^11^. The lipid compositions are as stated in the experimental conditions of the corresponding results. Short cantilevers (∼7 μm) with a nominal spring constant of 0.15 N m^−1^ were used (USC-F1.2-k0.15, NanoWorld) for all experiments. A minimal imaging force was applied by using a small set-point amplitude, typically 80% of the free amplitude (for 1–2 nm of free amplitude). The lipid bilayer was obtained by incubating LUVs (at a total lipid concentration of 0.5 mg ml^−1^) containing synthetic lipids (as stated in the paper) with or without lipid II (prepared as mentioned above) on top of freshly cleaved mica for 30–40 min. After the incubation period, the mica was cleaned gently using recording buffer (10 mM Tris–Cl, 100 mM NaCl, pH 7.2 with or without 1 mM CaCl_2_). Imaging was started on the lipid bilayer surface in recording buffer. Next, a concentrated plectasin solution was added to reach the desired final concentration of plectasin in the 40 µl AFM chamber. The results reported at varying plectasin concentrations (Fig. 5a) or varying H16A plectasin mutant concentrations (Fig. 5c) were performed on the same sample by adding additional plectasin (or the mutant) on the AFM sample chamber to reach the corresponding final concentration. All reported experiments were repeated for at least three times for each condition. Image analysis was done using Igor Pro version 6.22 (Wavemetrics) with RIBM script and ImageJ version 1.501 with minimal image corrections (tilt, drift and background corrections). The time stamps on HS-AFM videos are relative to plectasin addition into the liquid chamber. Image acquisition rate varied from 0.5 frames per second to 2 frames per second (see the figure/video legends).

### Reporting summary

Further information on research design is available in the Nature Portfolio Reporting Summary linked to this article.

### Supplementary information

Supplementary Information Supplementary Figs. 1–8, Tables 1–3, video captions and references.

Reporting Summary

Supplementary Video 1 HS-AFM video taken on a supported lipid bilayer composed of DOPC with 1% LII after addition of 1 µM plectasin in the absence of Ca^2+^. Imaging rate, 1 fps (frame per second).

Supplementary Video 2 HS-AFM video taken on a supported lipid bilayer composed of DOPC with 1% LII after addition of 1 µM plectasin in the presence of 1 mM Ca^2+^. Imaging rate, 0.2 fps.

Supplementary Video 3 HS-AFM video taken on a supported lipid bilayer composed of DOPC with 1% LII after addition of 1 µM plectasin in the presence of 1 mM Ca^2+^ and 10 mM EDTA. Imaging rate, 1 fps.

Supplementary Video 4 HS-AFM video taken on a supported lipid bilayer composed of DOPC/DOPG with 1% lipid II after addition of 1 µM plectasin in the absence of Ca^2+^. Imaging rate, 0.2 fps.

Supplementary Video 5 HS-AFM video taken on a supported lipid bilayer composed of DOPC/DOPG with 1% lipid II after addition of 1 µM plectasin in the presence of Ca^2+^. Imaging rate, 0.5 fps.

Supplementary Video 6 Zoomed in HS-AFM video taken on a supported lipid bilayer composed of DOPC/DOPG with 1% lipid II after addition of 1 µM plectasin in the absence of Ca^2+^. Formation of highly dynamic fibrils-like plectasin oligomers can be observed. Imaging rate, 5 fps.

Supplementary Video 7 Comparative video clips of surface dynamics and topography of plectasin covered lipid bilayer composed of DOPC/DOPG with 1% lipid II in the presence (left) and the absence (right) of Ca^2+^. Imaging rate, 0.5 fps.

### Source data

Source Data Fig. 1 NMR chemical shift derived values.

Source Data Fig. 2 NMR data.

Source Data Fig. 3 NMR relaxation data.

Source Data Fig. 4 NMR and AFM data.

Source Data Fig. 5 NMR, AFM and fluorescence spectroscopy data.

Source Data Extended Data Fig. 1 NMR chemical shift derived values.

Source Data Extended Data Fig. 3 NMR relaxation data.

Source Data Extended Data Fig. 8 Permeabilization assays—raw data.

Source Data Extended Data Fig. 9 NMR chemical shift derived values.

## Figures and Tables

**Extended Data Table 1:**
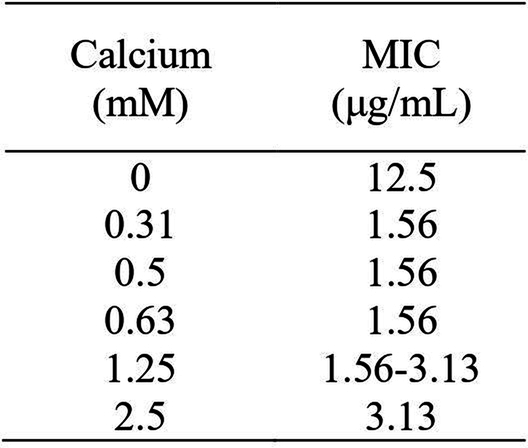
Antimicrobial activity assays of plectasin against S. simulans 22 at different calcium concentrations (Minimum inhibitory concentration)

## Data Availability

The NMR assignments of the plectasin–lipid II complex in different membrane environments have been deposited in the BMRB database (accession number 51880). Experimental solid-state NMR raw data are available via Zenodo at 10.5281/zenodo.7985263 (ref. ^55^). Source data are provided with this paper.

## References

[1] Lewis K (2020). The science of antibiotic discovery. Cell.

[2] Brown ED, Wright GD (2016). Antibacterial drug discovery in the resistance era. Nature.

[3] Cook, M. A. & Wright, G. D. The past, present, and future of antibiotics. *Sci. Transl. Med*. 10.1126/scitranslmed.abo7793 (2022). 10.1126/scitranslmed.abo7793 35947678

[4] Mygind PH (2005). Plectasin is a peptide antibiotic with therapeutic potential from a saprophytic fungus. Nature.

[5] Schneider T (2010). Plectasin, a fungal defensin, targets the bacterial cell wall precursor lipid II. Science.

[6] Ostergaard C, Sandvang D, Frimodt-Moller N, Kristensen HH (2009). High cerebrospinal fluid (CSF) penetration and potent bactericidal activity in CSF of NZ2114, a novel plectasin variant, during experimental pneumococcal meningitis. Antimicrob. Agents Chemother..

[7] Brinch KS (2010). Intracellular activity of the peptide antibiotic NZ2114: studies with *Staphylococcus aureus* and human THP-1 monocytes, and comparison with daptomycin and vancomycin. J. Antimicrob. Chemother..

[8] Zhang Y (2015). In vitro and in vivo characterization of a new recombinant antimicrobial peptide, MP1102, against methicillin-resistant *Staphylococcus aureus*. Appl. Microbiol. Biotechnol..

[9] Tenland E (2018). A novel derivative of the fungal antimicrobial peptide plectasin is active against *Mycobacterium tuberculosis*. Tuberculosis.

[10] Brinch KS (2009). Plectasin shows intracellular activity against *Staphylococcus aureus* in human THP-1 monocytes and in a mouse peritonitis model. Antimicrob. Agents Chemother..

[11] Shukla R (2022). Teixobactin kills bacteria by a two-pronged attack on the cell envelope. Nature.

[12] Shukla R (2020). Mode of action of teixobactins in cellular membranes. Nat. Commun..

[13] Shukla R (2023). An antibiotic from an uncultured bacterium binds to an immutable target. Cell.

[14] Wang Y, Jardetzky O (2009). Probability-based protein secondary structure identification using combined NMR chemical-shift data. Protein Sci..

[15] Jekhmane S (2019). Shifts in the selectivity filter dynamics cause modal gating in K+ channels. Nat. Commun..

[16] Gullion T, Schaefer J (1989). Rotational-echo double-resonance NMR. J. Magn. Reson..

[17] Baldus M, Meier BH (1996). Total correlation spectroscopy in the solid state. The use of scalar couplings to determine the through-bond connectivity. J. Magn. Reson. A.

[18] Hackel MA (2019). Reproducibility of broth microdilution MICs for the novel siderophore cephalosporin, cefiderocol, determined using iron-depleted cation-adjusted Mueller–Hinton broth. Diagn. Microbiol. Infect. Dis..

[19] Ho SW (2007). Effect of divalent cations on the structure of the antibiotic daptomycin. Eur. Biophys. J..

[20] van Dam V (2007). Transmembrane transport of peptidoglycan precursors across model and bacterial membranes. Mol. Microbiol..

[21] Etzkorn M, Böckmann A, Lange A, Baldus M (2004). Probing molecular interfaces using 2D magic-angle-spinning NMR on protein mixtures with different uniform labeling. J. Am. Chem. Soc..

[22] Visscher KM (2017). Supramolecular organization and functional implications of K^+^ channel clusters in membranes. Angew. Chem. Int. Ed..

[23] Ni QZ (2013). High frequency dynamic nuclear polarization. Acc. Chem. Res..

[24] Kaplan M (2016). EGFR dynamics change during activation in native membranes as revealed by NMR. Cell.

[25] Li S, Hong M (2011). Protonation, tautomerization, and rotameric structure of histidine: a comprehensive study by magic-angle-spinning solid-state NMR. J. Am. Chem. Soc..

[26] Kodera N, Yamamoto D, Ishikawa R, Ando T (2010). Video imaging of walking myosin V by high-speed atomic force microscopy. Nature.

[27] Maity S (2020). Caught in the act: mechanistic insight into supramolecular polymerization-driven self-replication from real-time visualization. J. Am. Chem. Soc..

[28] Melcrová A (2023). Lateral membrane organization as target of an antimicrobial peptidomimetic compound. Nat. Commun..

[29] Medeiros-Silva J (2018). High-resolution NMR studies of antibiotics in cellular membranes. Nat. Commun..

[30] Hasper HE (2006). An alternative bactericidal mechanism of action for lantibiotic peptides that target lipid II. Science.

[31] Essig A (2014). Copsin, a novel peptide-based fungal antibiotic interfering with the peptidoglycan synthesis. J. Biol. Chem..

[32] Cudic P (2002). Complexation of peptidoglycan intermediates by the lipoglycodepsipeptide antibiotic ramoplanin: minimal structural requirements for intermolecular complexation and fibril formation. Proc. Natl Acad. Sci. USA.

[33] Müller A (2016). Daptomycin inhibits cell envelope synthesis by interfering with fluid membrane microdomains. Proc. Natl Acad. Sci. USA.

[34] Yang H, Chen KH, Nowick JS (2016). Elucidation of the teixobactin pharmacophore. ACS Chem. Biol..

[35] Horton RM, Cai ZL, Ho SN, Pease LR (1990). Gene splicing by overlap extension: tailor-made genes using the polymerase chain reaction. Biotechniques.

[36] de Jong RN, Daniëls MA, Kaptein R, Folkers GE (2006). Enzyme free cloning for high throughput gene cloning and expression. J. Struct. Funct. Genomics.

[37] Breukink E (2003). Lipid II is an intrinsic component of the pore induced by nisin in bacterial membranes. J. Biol. Chem..

[38] Danilov LL, Druzhinina TN, Kalinchuk NA, Maltsev SD, Shibaev VN (1989). Polyprenyl phosphates: synthesis and structure–activity relationship for a biosynthetic system of *Salmonella* anatum O-specific polysaccharide. Chem. Phys. Lipids.

[39] Cochrane RVK (2020). From plant to probe: semi-synthesis of labelled undecaprenol analogues allows rapid access to probes for antibiotic targets. Chem. Commun..

[40] Rouser G, Siakotos AN, Fleischer S (1966). Quantitative analysis of phospholipids by thin-layer chromatography and phosphorus analysis of spots. Lipids.

[41] Hope MJ, Bally MB, Webb G, Cullis PR (1985). Production of large unilamellar vesicles by a rapid extrusion procedure: characterization of size distribution, trapped volume and ability to maintain a membrane potential. Biochim. Biophys. Acta.

[42] Sauvée C (2013). Highly efficient, water-soluble polarizing agents for dynamic nuclear polarization at high frequency.. Angew. Chem. Int. Ed..

[43] Weingarth M, Bodenhausen G, Tekely P (2009). Low-power decoupling at high spinning frequencies in high static fields. J. Magn. Reson..

[44] Weingarth M, Tekely P, Bodenhausen G (2008). Efficient heteronuclear decoupling by quenching rotary resonance in solid-state NMR. Chem. Phys. Lett..

[45] Weingarth M, Bodenhausen G, Tekely P (2010). Broadband magnetization transfer using moderate radio-frequency fields for NMR with very high static fields and spinning speeds. Chem. Phys. Lett..

[46] Weingarth M, Demco DE, Bodenhausen G, Tekely P (2009). Improved magnetization transfer in solid-state NMR with fast magic angle spinning. Chem. Phys. Lett..

[47] Fung BM, Khitrin AK, Ermolaev K (2000). An improved broadband decoupling sequence for liquid crystals and solids. J. Magn. Reson..

[48] Medeiros-Silva J (2016). (1) H-detected solid-state NMR studies of water-inaccessible proteins in vitro and in situ. Angew. Chem. Int. Ed. Engl..

[49] Doherty T, Hong M (2009). 2D 1H-31P solid-state NMR studies of the dependence of inter-bilayer water dynamics on lipid headgroup structure and membrane peptides. J. Magn. Reson..

[50] Gullion T (1998). Introduction to rotational-echo, double-resonance NMR. Concept. Magn. Reson..

[51] Vinod Chandran C, Madhu PK, Kurur ND, Brauniger T (2008). Swept-frequency two-pulse phase modulation (SWf-TPPM) sequences with linear sweep profile for heteronuclear decoupling in solid-state NMR. Magn. Reson. Chem..

[52] van Beekveld RAM (2022). Specific lipid studies in complex membranes by solid‐state NMR spectroscopy. Chemistry.

[53] Peng JW, Thanabal V, Wagner G (1991). 2D heteronuclear NMR measurements of spin-lattice relaxation times in the rotating frame of X nuclei in heteronuclear HX spin systems. J. Magn. Reson..

[54] Jarmoskaite I, AlSadhan I, Vaidyanathan PP, Herschlag D (2020). How to measure and evaluate binding affinities. Elife.

[55] Derks, M. G. N. & Weingarth, M. ssNMR raw data ‘Host defence peptide plectasin targets bacterial cell wall precursor lipid II by a calcium-sensitive supramolecular mechanism’. *Zenodo*10.5281/zenodo.7985263 (2023). 10.1038/s41564-024-01696-9 PMC11222147 38783023

