# Supplementary material for: Host defence peptide plectasin targets bacterial cell wall precursor lipid II by a calcium-sensitive supramolecular mechanism
Source: Nat Microbiol. 2024 May 23;9(7):1778–91. doi: 10.1038/s41564-024-01696-9 (PMC11222147; doi:10.1038/s41564-024-01696-9)
Supplement: Supplementary file 1 — Supplementary Figs. 1–8, Tables 1–3, video captions and references. [file 41564_2024_1696_MOESM1_ESM.pdf]

nature microbiology

Supplementary information

https://doi.org/10.1038/s41564-024-01696-9

Host defence peptide plectasin targets bacterial cell wall precursor lipid II by a calcium-sensitive supramolecular mechanism

In the format provided by the authors and unedited

Table of Contents

Supplementary Fig 1: ssNMR chemical shift assignments of lipid II in complex with plectasin 02
Supplementary Fig 2: DNP ssNMR confirms plectasin oligomerization 03
Supplementary Fig 3: Influence of membrane composition and bivalent cations 05
Supplementary Fig 4: *S. simulans 22* grows in bivalent cation-deprived medium 07
Supplementary Fig 5: Expression and purification of plectasin in *SHuffle E. coli* 08
Supplementary Fig 6: Characterization of UndP-N3 09
Supplementary Fig 7: Synthesis of Und-NBD-Lipid II 10
Supplementary Fig 8: Uncropped gels 11
Supplementary Table 1: Activity assays of plectasin mutants 12
Supplementary Table 2: Solution NMR chemical shifts of free plectasin 13
Supplementary Table 3: ssNMR chemical shifts of plectasin bound to Lipid II in membranes 14
Supplementary Videos 15
References 16

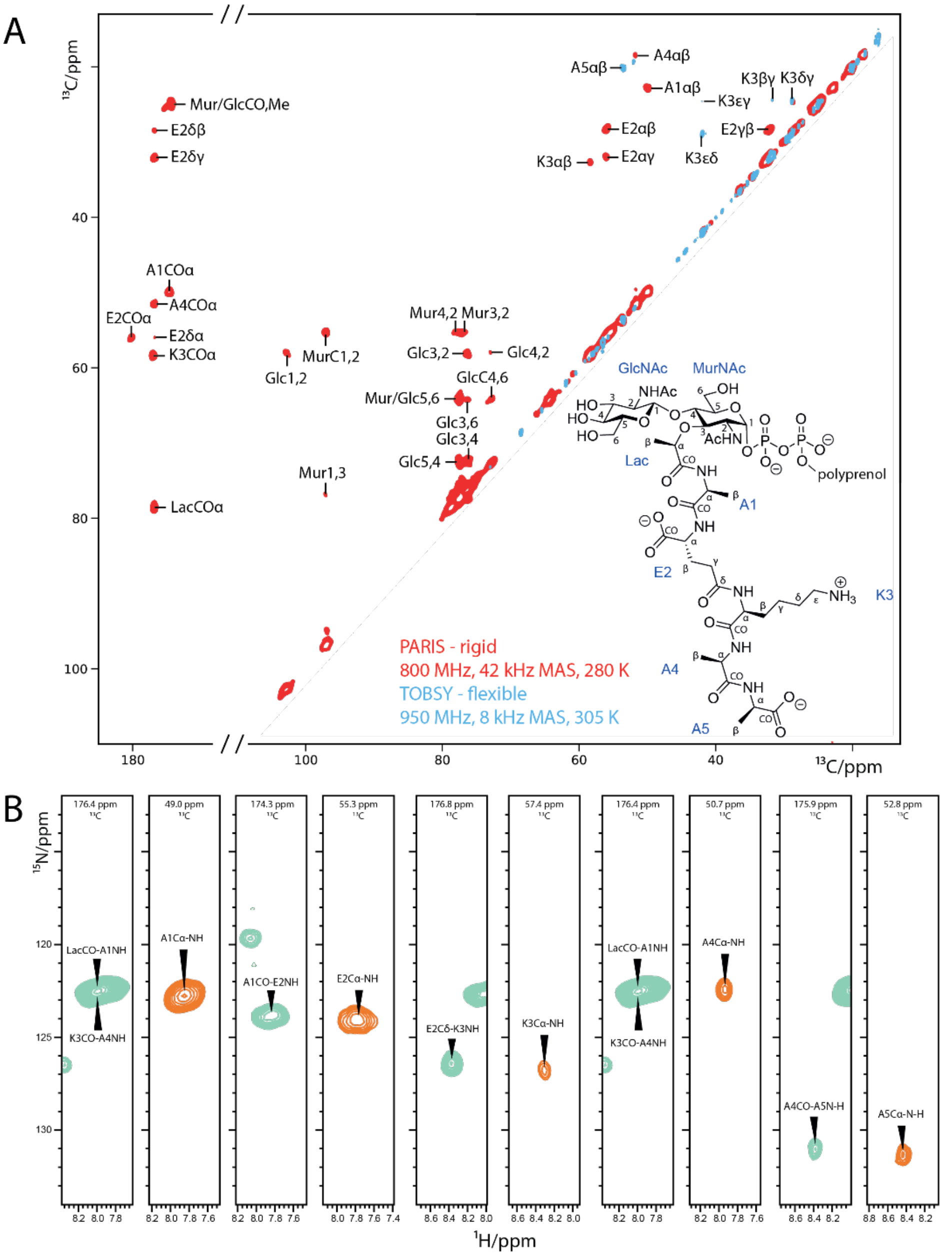

Supplementary Figure 1. ssNMR chemical shift assignments of lipid II in complex with plectasin

A) Chemical shift assignments of Lipid II in complex with plectasin. Overlay of 2D $^{13}C^{13}C$ PARIS-xy (red) and TOBSY[1] (blue) spectra, sensitive to rigid and mobile regions respectively. The PARIS spectrum was recorded at a magnetic field of 800 MHz (^{1}H frequency), 42 kHz MAS and a sample temperature of 280 K. The TOBSY spectrum was recorded at a magnetic field of 950 MHz (^{1}H frequency), 8 kHz MAS and a sample temperature of 305 K. **B)** Strip plots of 3D CONH (teal) and CαNH (orange) spectra used for the backbone assignments of the pentapeptide of Lipid II. The CONH was recorded at 700 MHz (^{1}H frequency), 60 kHz MAS and a sample temperature of 305 K. The CαNH was recorded with 50% NUS at 800 MHz (^{1}H frequency), 60 kHz MAS and a sample temperature of 305 K. In contrast to the ^{13}C detected experiments in A, both A4 and A5 were detectable in these dipolar coupling-based experiments due to the higher heteronuclear dipolar couplings with protons and the increased sensitivity of proton detection.

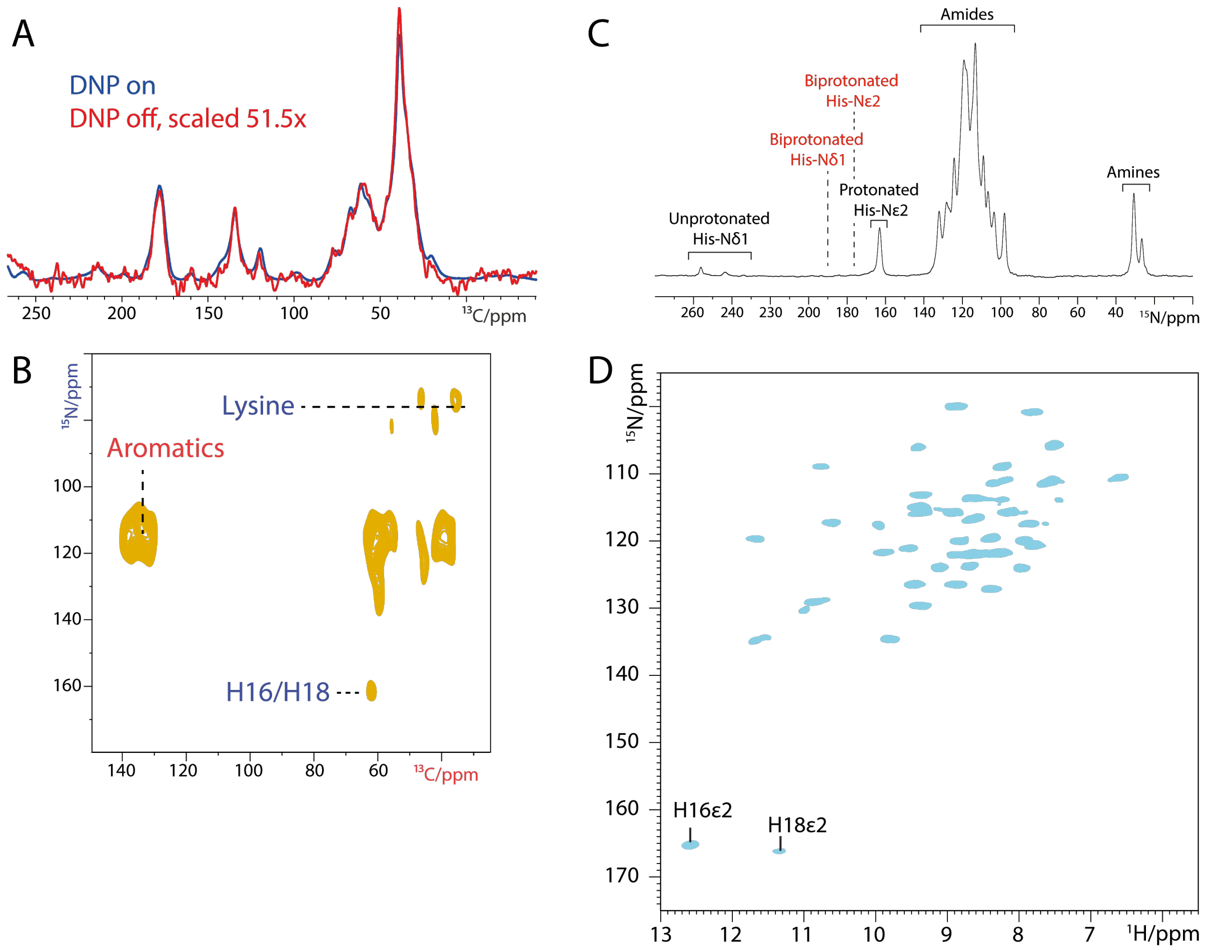

Supplementary Figure 2. 'FRET-like' DNP-ssNMR confirms that plectasin oligomerizes upon Lipid II binding in membranes.

A,B) Plectasin oligomerizes upon Lipid II binding in membranes. The plectasin-plectasin intermolecular interface was investigated using a FRET-like ssNMR experiment[2]. For this experimental setup, equimolar quantities of ^{13}C- and ^{15}N-labelled plectasin where mixed and then added to Lipid-II doped DOPC liposomes. Magnetization transferred from ^{15}N to ^{13}C can then only be established via intermolecular contacts between ^{13}C- and ^{15}N-labelled molecules. Since this transfer is short-ranged (approximately 0.5 nm), plectasin molecules need to tightly interact to detect ^{15}N to ^{13}C magnetization transfer. In order to exclude that intermolecular transfer relates to spurious aggregation due to crowding on the membrane surface, we acquired N(HH)C-spectra[3] using low (0.5 mol%) Lipid II concentration (see methods section for more details). As the low Lipid II concentration very strongly reduce the spectral sensitivity, we used DNP-signal enhancement[2,4,5] to compensate. AMUPol[6] was used as polarization agent. **A)** Enhancement by DNP of plectasin at low (0.5 mol%) lipid II concentration. Spectra were acquired at 400 MHz (^{1}H frequency), 8 kHz MAS and 100 K sample temperature with the microwave source turned off (red) or on (blue). Spectrum without DNP enhancement was scaled vertically by a factor of 51.53. **B)** 2D NHHC experiment acquired under DNP. Spectrum was acquired at 400 MHz (^{1}H frequency), 8 kHz MAS and 100 K sample temperature using a ^{1}H-^{1}H mixing time of 200 µs. **C,D)** While no experimental data on the histidine-sidechains were reported, previous studies in micelles[7] conjectured that the H18 would be protonated and would interact with the anionic pentapeptide of Lipid II. This is clearly refuted by our NMR studies in membranes, in which we can directly and clearly determine the protonation states. **C**) ssNMR^{15}N-cp spectrum of ^{15}N-labeled plectasin in complex with lipid II in DOPG

membranes. Spectrum was acquired at 800 MHz (^{1}H frequency), 60 kHz MAS and a sample temperature of 305 K. A long ^{1}H-^{15}N cross-polarization contact time of 4.5 ms was used in order to observe the unprotonated imidazole nitrogens. Literature chemical shifts of cationic bi-protonated histidine sidechains are shown in red[8]. The data unambiguously show that both H16 and H18 sidechains are neutral. **D**) ssNMR ^{1}H-detected 2D NH spectrum of plectasin in complex with Lipid II in DOPC membranes using a wide spectral width in the ^{15}N dimension displaying the unfolded peaks of the histidine sidechains. Spectrum was acquired at 1200 MHz (^{1}H frequency), 60 kHz MAS and a sample temperature of 290 K.

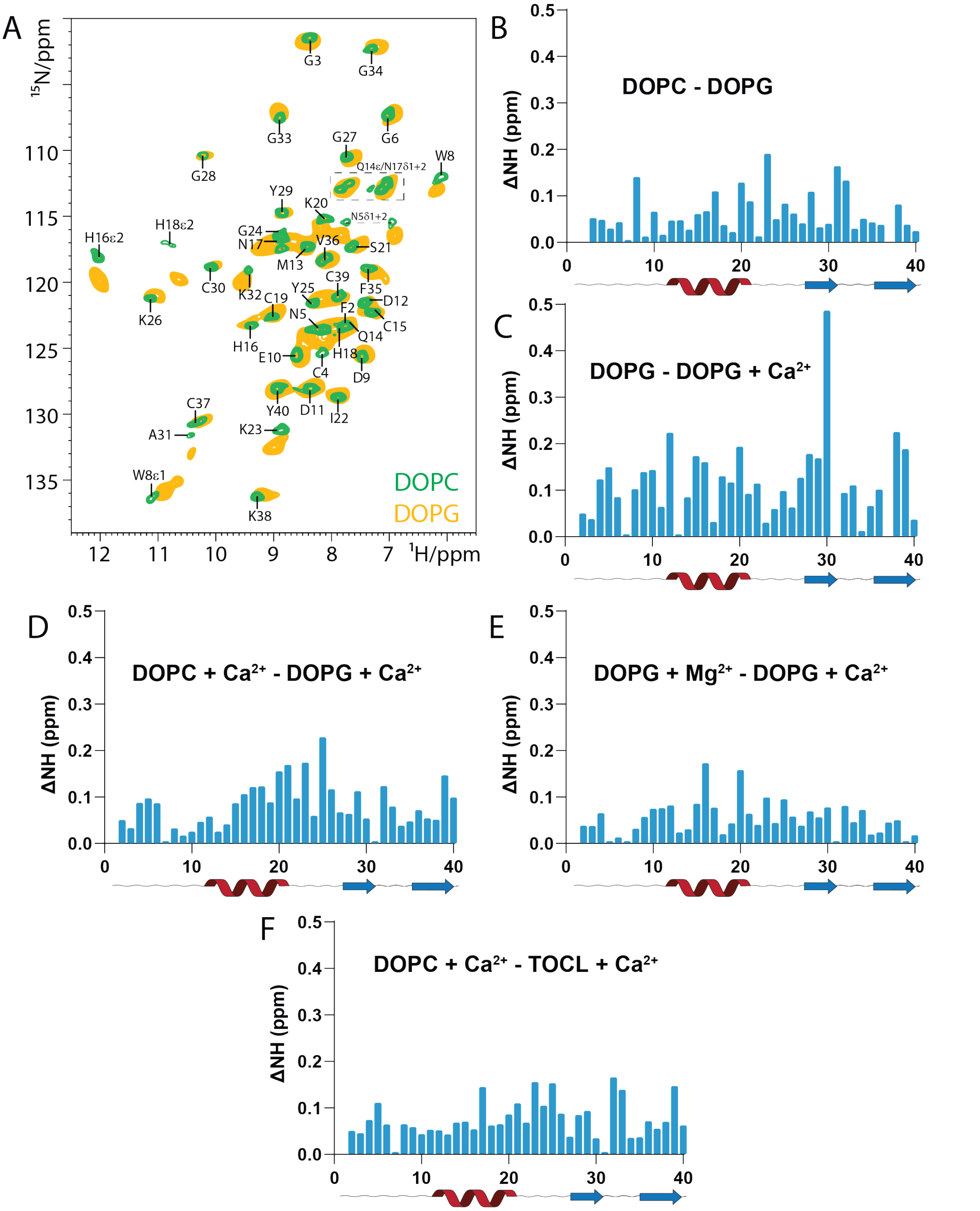

Supplementary Figure 3. Influence of membrane composition and bivalent cations on the plectasin-Lipid II complex.

ssNMR amide fingerprints and corresponding CSPs of plectasin in complex with Lipid II in either DOPC or anionic DOPG membranes and with or without the addition of Ca^{2+} or Mg^{2+}. Spectra were recorded at a magnetic field of 28.2 T (1200 MHz ^{1}H frequency) at 60 kHz MAS and a sample temperature of 305 K, with the exception of DOPG without calcium, which was recorded at 16.4 T (700 MHz ^{1}H frequency). **A)** Example overlay of NH fingerprint of ^{15}N-plectasin in complex with Lipid II in zwitterionic (DOPC, green) and anionic (DOPG, yellow)

lipid vesicles. Note that H16ε2 and H18ε2 similar ^{15}N and ^{1}H chemical shifts in both spectra, but signals are shifted due to different spectral widths in the indirect (^{15}N) dimension and resulting differential spectral backfolding. **B)** CSPs of DOPC and DOPG membranes both without Ca^{2+}. **C)** CSPs of addition of Ca^{2+} in DOPG membranes. **D)** CSPs of DOPC and DOPG membranes both in the presence of Ca^{2+} **E)** CSPs between addition of Mg^{2+} or Ca^{2+} in anionic DOPG membranes. **F**) CSPs between DOPC and TOCL membranes both in the presence of Ca^{2+}

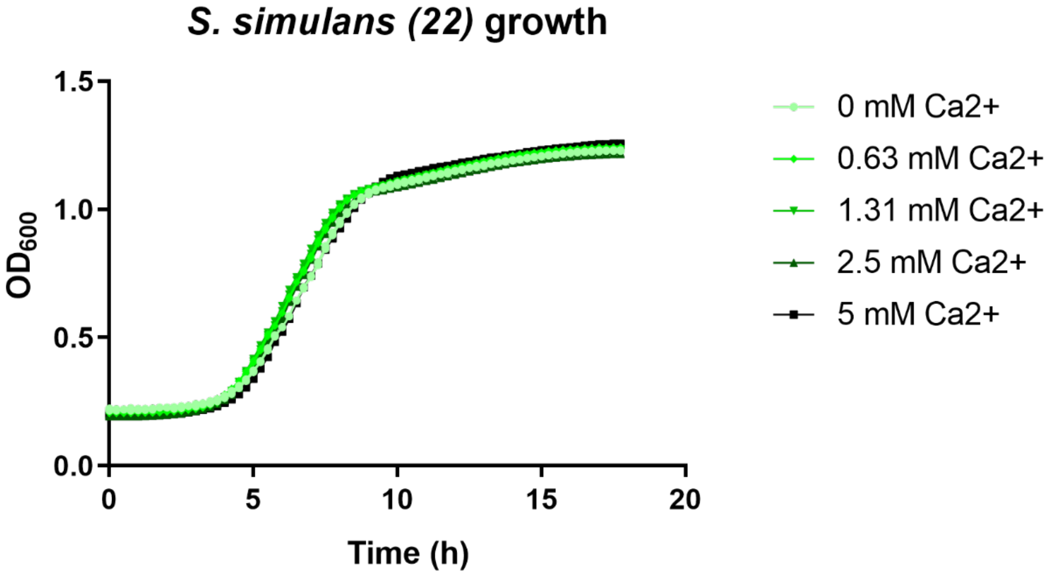

Supplementary Figure 4. Controls for the growth of *S. simulans* 22 in bivalent cation-deprived medium

Experimental procedure is described in the methods section. Growth was followed by monitoring the optical density at 600 nm for 18 h in a plate reader. *S. simulans* grew well at all used Ca^{2+} concentrations.

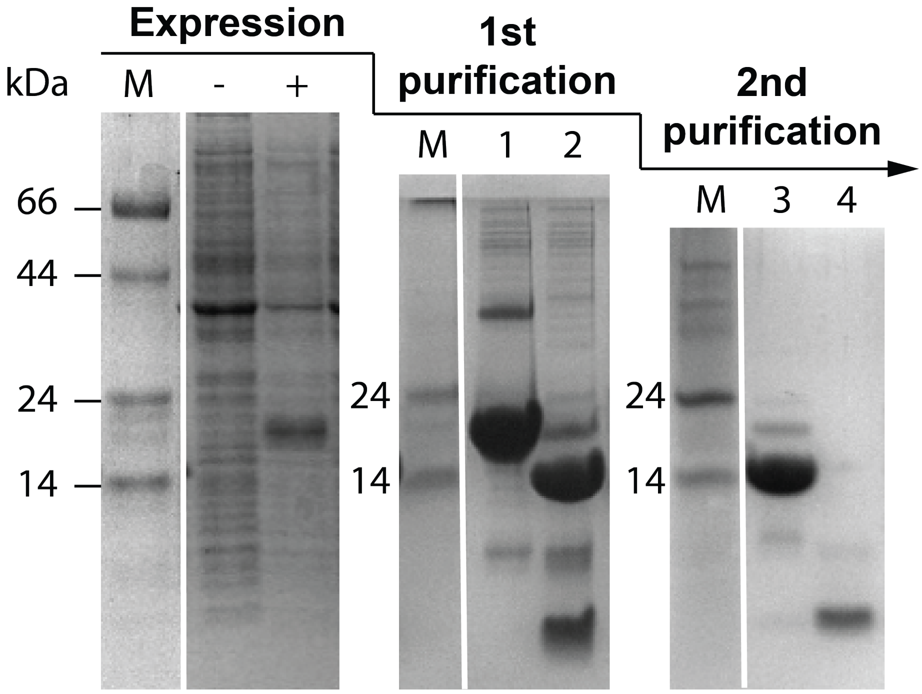

Supplementary Figure 5. Expression and purification of plectasin in SHuffle E. coli.

Expression: SDS-PAGE analysis of protein expression induced with 0.5 mM IPTG for 4 hours at 37°C. Lane M contains protein marker, lane – negative control where no expression was induced and lane + the overexpression product of the petSUMO – plectasin construct (± 18 kDa). **1st purification** SDS-PAGE analysis of the purification of the recombinant fusion protein. Lane M contains marker, lane 1 contains the fusion protein after purification by affinity chromatography, lane 2 shows cleavage of SUMO from plectasin by SUMO protease (Ulp1). **2nd purification** SDS page analysis of purification of plectasin. Lane M contains marker, lane 3 and 4 contain fractions collected after gel filtration containing SUMO (±14 kDa) and plectasin (±4.4 kDa), respectively. Uncropped gels are presented in Supplementary Figure 8.

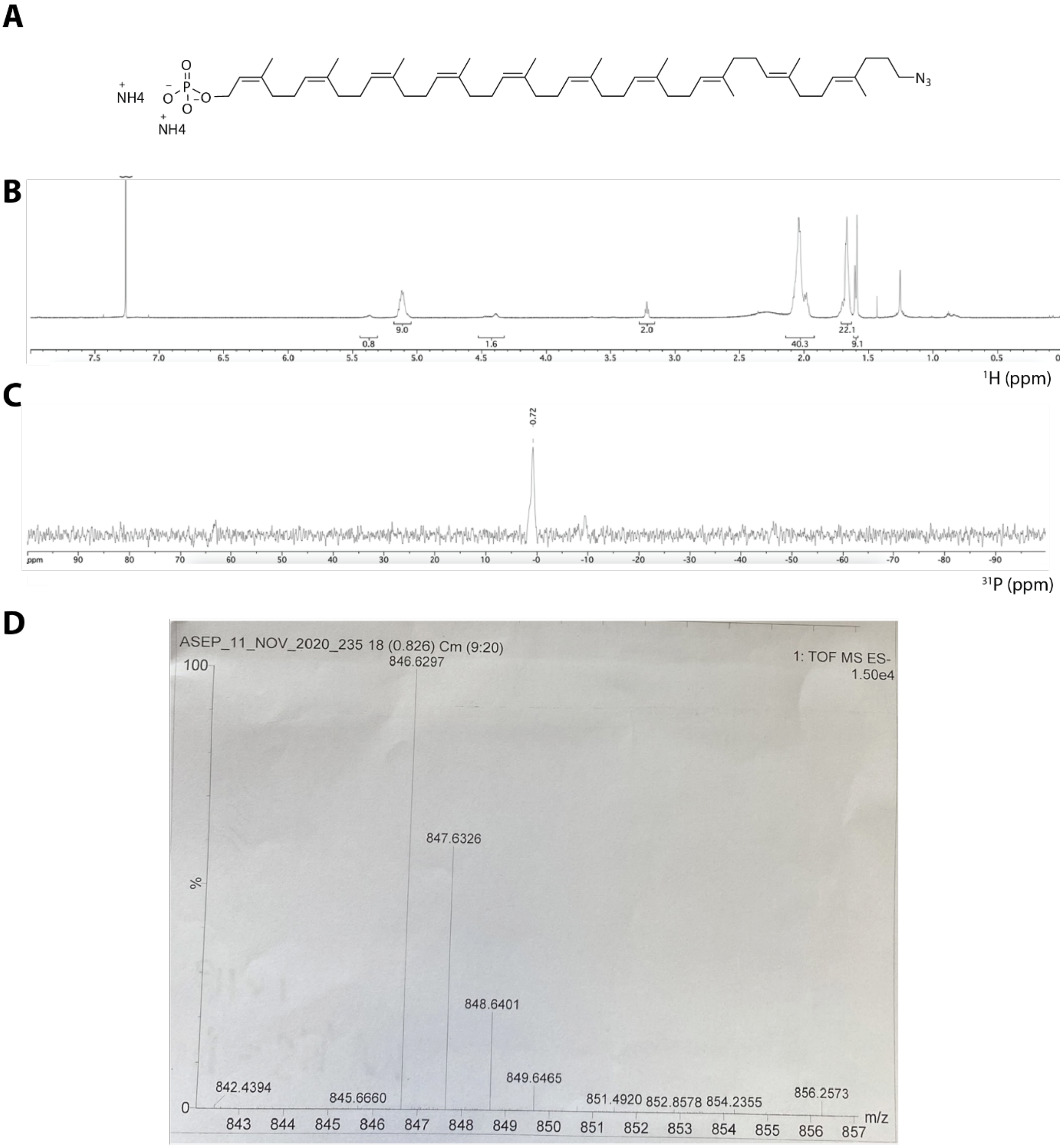

Supplementary Figure 6. Characterization of UndP-N$_3$.

A) Structure of UndP-N$_3$ **B**) ^{1}H-NMR (400 MHz, CDCl$_3$): δ 5.43-5.33 (1H, m, α-CH), 5.19-5.04 (9H, m, 9 x alkene C-H), 4.50-4.34 (2H, m, α-CH$_2$), 3.21 (1H, t, *J* 6.9, N$_3$-CH$_2$), 2.14-1.94 (40H, m, 20 x CH$_2$), 1.71-1.63 (21H, m, 7 x *cis*-alkene CH$_3$), 1.61-1.57 (9H, m, 3 x *E*-alkene CH$_3$) **C**) ^{31}P-NMR (162 MHz, CDCl$_3$): δ 0.72 (bs) **D**) ESI-HRMS (m/z): [M-H]$^-$ calcd for C$_{52}$H$_{85}$N$_3$O$_4$P: 846.6277; found: 846.6297

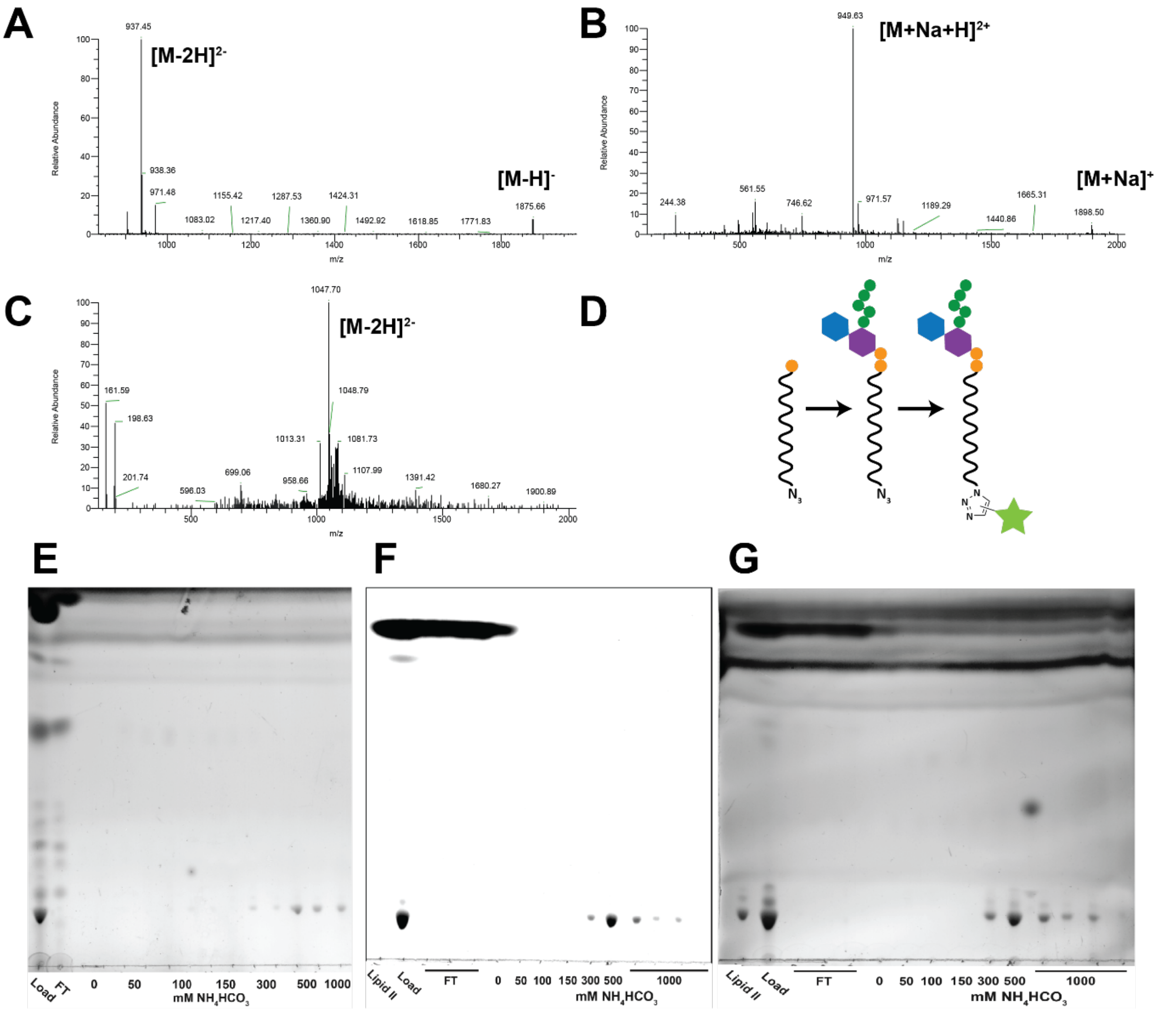

Supplementary Figure 7. Synthesis of Und-NBD-Lipid II.

Mass spectrometry traces of **A)** Und-N_3-Lipid II (neg. mode). Calc. $[M-2H]^{2-}$ = 937.02, $[M-H]^-$ = 1875.03, **B**) Und-N_3-Lipid II (pos. mode). Calc. $[M+Na+H]^{2+}$ = 950.015, $[M+Na]^+$ = 1899.03, **C**) Und-NBD-Lipid II (neg. mode). Calc. $[M-2H]^{2-}$ = 1046.53 and **D**) Schematic summary of synthesis. **E**), **F**) & **G**) TLC analysis of the purification on DEAE cellulose of **E)** Und-N_3-Lipid II, **F)** Und-NBD-Lipid II (unstained) and **G)** Und-NBD-Lipid II stained with iodine.

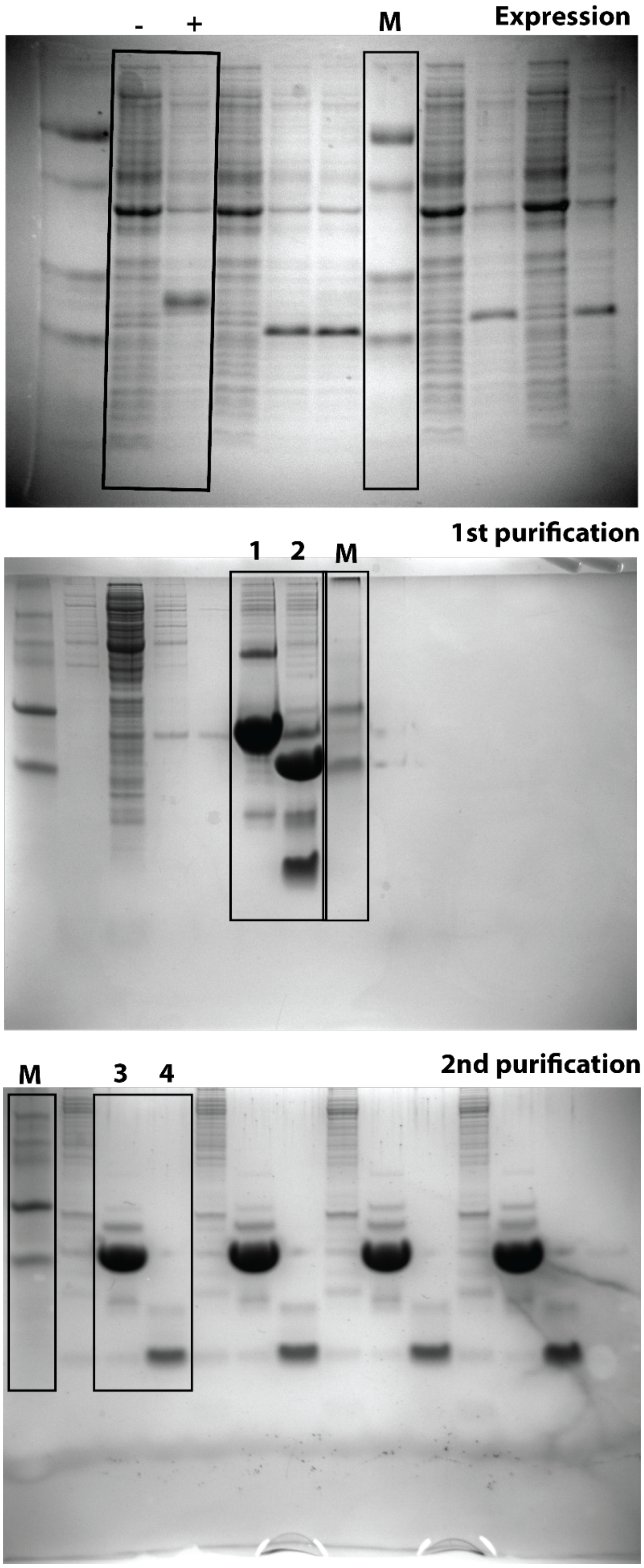

Supplementary Figure 8. Uncropped gels of plectasin expression and purification.

The boxes indicate the cutouts that are shown in Supplementary Figure 5, using identical labeling for the lanes.

Supplementary Table 1. Activity assays of plectasin mutants (Minimum inhibitory concentration in μg/mL)

Drug/Strain	*S. simulans*	*S. pneumoniae serotype 8*	*S. pneumoniae ATCC6305*	*S. pyogenes* M3
Plectasin wt	0,78	1,56	≤0.78	≤0,012
H16A	>50			
H18A	>50			
I22A		>25		>25
I22D	>50			
I22F	>50			
I22K	>50		>50	
I22L	1,56			
I22V	1,56			
Y25F	1,56		3,125	
Y25H	≤0.78			
Y25I	6,25			
Y25L	3,13			
Y25V	6,25			
K26A	3,13		3,13	
K26R	≤0.78		1,56	
Y29A	12,5		>50	
Y29F	1,56		≤0.78	
Y29R	1,56		1,56	
Y40R	>50		>50	
Y40F	1,56		0,78	
Vancomycin	0,78		<0.78	

Supplementary Table 2. Solution NMR chemical shifts of free plectasin (in ppm).

Residue	H^N	N	Cα	Cβ
G1				
F2			59.03	39.32
G3	7.86	101.40	44.35	
C4	7.52	123.24	52.87	34.79
N5	8.11	121.58	51.93	39.63
G6	7.19	110.13	42.66	
P7			64.21	31.07
W8	7.28	117.10	56.61	27.81
D9	7.59	124.01	53.78	40.66
E10	8.08	122.29	55.31	30.97
D11	8.09	125.82	52.31	40.95
D12	8.35	124.13	57.56	41.43
M13	8.13	118.20	57.49	31.31
Q14	7.57	121.63	58.93	28.62
C15	7.29	122.00	58.15	35.67
H16	8.91	122.83	60.77	31.84
N17	8.29	115.70	55.86	37.94
H18	7.90	123.07	59.80	29.47
C19	8.61	120.84	58.51	37.10
K20	7.73	115.11	58.06	31.51
S21	7.39	115.65	59.39	63.87
I22	7.51	126.56	60.70	37.94
K23	7.99	126.79	58.29	34.46
G24			44.83	
Y25	7.65	120.64	56.68	38.65
K26	10.50	121.55	55.89	34.74
G27	7.21	107.65	45.61	
G28	8.61	108.47	47.03	
Y29	8.33	116.39	55.94	40.09
C30	9.08	118.47	52.92	36.65
A31	9.50	129.93	50.72	22.58
K32	8.84	119.81	56.62	
G33			45.84	
G34	7.31	105.04	44.51	
F35	7.13	117.08	60.25	41.30
V36	7.63	118.91	60.66	35.48
C37	8.69	127.85	55.66	35.64
K38	8.92	136.75	54.59	34.00
C39	7.76	121.99	51.04	34.60
Y40	8.24	127.13	58.31	40.23

Supplementary Table 3. ssNMR chemical shifts of plectasin bound to Lipid II in membranes (in ppm).

Residue	H^N	N	Cα	Cβ
**G1**				
**F2**	7.72	123.47	59.54	36.39
**G3**	8.34	101.44	44.59	
**C4**	8.10	124.73	52.86	33.43
**N5**	8.18	123.85	52.07	39.45
**G6**	6.90	107.32	41.77	
**P7**			64.88	31.06
**W8**	6.09	117.10	56.54	28.04
**D9**	7.41	125.39	53.82	40.22
**E10**	8.56	125.19	54.98	27.89
**D11**	8.32	128.12	51.14	39.42
**D12**	7.32	121.46	57.82	43.29
**M13**	8.39	117.30	56.70	30.22
**Q14**	7.71	123.19	59.16	27.99
**C15**	7.24	122.20	58.41	36.54
**H16**	9.41	123.36	61.03	31.72
**N17**	8.82	116.97	55.60	37.59
**H18**	7.81	123.18	59.16	29.71
**C19**	8.95	122.31	58.44	36.63
**K20**	8.10	115.19	58.04	31.68
**S21**	7.53	117.28	59.23	64.48
**I22**	7.80	128.45	61.14	39.77
**K23**	8.79	131.07	58.93	32.34
**G24**	8.83	116.44	44.43	
**Y25**	8.26	121.54	58.49	40.66
**K26**	11.04	120.90	54.54	35.34
**G27**	7.66	110.39	46.56	
**G28**	10.08	110.06	47.28	
**Y29**	8.77	114.67	56.71	39.89
**C30**	9.97	118.59	52.83	36.97
**A31**	10.36	132.28	50.35	23.40
**K32**	9.35	118.43	56.42	34.89
**G33**	8.85	107.73	46.45	
**G34**	7.21	102.17	44.23	
**F35**	7.25	119.09	61.06	41.75
**V36**	8.12	118.24	60.16	35.98
**C37**	10.14	130.40	55.81	35.22
**K38**	9.16	136.35	55.16	35.84
**C39**	7.84	121.02	50.76	35.84
**Y40**	8.87	128.09	58.94	40.73

Supplementary Movies

Supplementary Movie 1:

HS-AFM movie taken on a supported lipid bilayer composed of DOPC with 1% LII after addition of 1 µM plectasin in absence of Ca^{2+}. Imaging rate : 1 fps (frame per second).

Supplementary Movie 2:

HS-AFM movie taken on a supported lipid bilayer composed of DOPC with 1% LII after addition of 1 µM plectasin in presence of 1 mM Ca^{2+}. Imaging rate : 0.2 fps

Supplementary Movie 3:

HS-AFM movie taken on a supported lipid bilayer composed of DOPC with 1% LII after addition of 1 µM plectasin in presence of 1 mM Ca^{2+} and 10 mM EDTA. Imaging rate : 1 fps

Supplementary Movie 4:

HS-AFM movie taken on a supported lipid bilayer composed of DOPC/DOPG with 1 % Lipid II after addition of 1 µM plectasin in absence of Ca^{2+}. Imaging rate : 0.2 fps.

Supplementary Movie 5:

HS-AFM movie taken on a supported lipid bilayer composed of DOPC/DOPG with 1 % Lipid II after addition of 1 µM plectasin in presence of Ca^{2+}. Imaging rate : 0.5 fps.

Supplementary Movie 6:

Zoomed in HS-AFM movie taken on a supported lipid bilayer composed of DOPC/DOPG with 1 % Lipid II after addition of 1 µM plectasin in absence of Ca^{2+}. Formation of highly dynamic fibrils-like plectasin oligomers can be observed. Imaging rate : 5 fps.

Supplementary Movie 7:

Comparative movie clips of surface dynamics and topography of plectasin covered lipid bilayer composed of DOPC/DOPG with 1 % Lipid II in presence (left) and absence (right) of Ca^{2+}. Imaging rate: 0.5 fps.

References

1 Baldus, M. & Meier, B. H. Total Correlation Spectroscopy in the Solid State. The Use of Scalar Couplings to Determine the Through-Bond Connectivity. *Journal of Magnetic Resonance, Series A* **121**, 65-69, doi:https://doi.org/10.1006/jmra.1996.0137 (1996).

2 Visscher, K. M. *et al.* Supramolecular Organization and Functional Implications of K+Channel Clusters in Membranes. *Angewandte Chemie International Edition* **56**, 13222-13227, doi:10.1002/anie.201705723 (2017).

3 Etzkorn, M., Böckmann, A., Lange, A. & Baldus, M. Probing Molecular Interfaces Using 2D Magic-Angle-Spinning NMR on Protein Mixtures with Different Uniform Labeling. *Journal of the American Chemical Society* **126**, 14746-14751, doi:10.1021/ja0479181 (2004).

4 Ni, Q. Z. *et al.* High Frequency Dynamic Nuclear Polarization. *Accounts of Chemical Research* **46**, 1933-1941, doi:10.1021/ar300348n (2013).

5 Kaplan, M. *et al.* EGFR Dynamics Change during Activation in Native Membranes as Revealed by NMR. *Cell* **167**, 1241-1251.e1211, doi:10.1016/j.cell.2016.10.038 (2016).

6 Sauvée, C. *et al.* Highly Efficient, Water-Soluble Polarizing Agents for Dynamic Nuclear Polarization at High Frequency. *Angewandte Chemie International Edition* **52**, 10858-10861, doi:10.1002/anie.201304657 (2013).

7 Schneider, T. *et al.* Plectasin, a Fungal Defensin, Targets the Bacterial Cell Wall Precursor Lipid II. *Science* **328**, 1168-1172, doi:10.1126/science.1185723 (2010).

8 Li, S. & Hong, M. Protonation, Tautomerization, and Rotameric Structure of Histidine: A Comprehensive Study by Magic-Angle-Spinning Solid-State NMR. *Journal of the American Chemical Society* **133**, 1534-1544, doi:10.1021/ja108943n (2011).
