# Supplementary material for: Host defence peptide plectasin targets bacterial cell wall precursor lipid II by a calcium-sensitive supramolecular mechanism
Source: Nat Microbiol. 2024 May 23;9(7):1778–91. doi: 10.1038/s41564-024-01696-9 (PMC11222147; doi:10.1038/s41564-024-01696-9)
Supplement: Supplementary file 2 — Reporting Summary [file 41564_2024_1696_MOESM2_ESM.pdf]

nature portfolio

Corresponding author(s): Markus Weingarth

Last updated by author(s): Mar 12, 2024

Reporting Summary

Nature Portfolio wishes to improve the reproducibility of the work that we publish. This form provides structure for consistency and transparency in reporting. For further information on Nature Portfolio policies, see our Editorial Policies and the Editorial Policy Checklist.

Statistics

For all statistical analyses, confirm that the following items are present in the figure legend, table legend, main text, or Methods section.

n/a	Confirmed	
☐	☒	The exact sample size (*n*) for each experimental group/condition, given as a discrete number and unit of measurement
☒	☐	A statement on whether measurements were taken from distinct samples or whether the same sample was measured repeatedly
☐	☒	The statistical test(s) used AND whether they are one- or two-sided<br>*Only common tests should be described solely by name; describe more complex techniques in the Methods section.*
☒	☐	A description of all covariates tested
☒	☐	A description of any assumptions or corrections, such as tests of normality and adjustment for multiple comparisons
☐	☒	A full description of the statistical parameters including central tendency (e.g. means) or other basic estimates (e.g. regression coefficient) AND variation (e.g. standard deviation) or associated estimates of uncertainty (e.g. confidence intervals)
☐	☒	For null hypothesis testing, the test statistic (e.g. *F*, *t*, *r*) with confidence intervals, effect sizes, degrees of freedom and *P* value noted<br>*Give P values as exact values whenever suitable.*
☒	☐	For Bayesian analysis, information on the choice of priors and Markov chain Monte Carlo settings
☒	☐	For hierarchical and complex designs, identification of the appropriate level for tests and full reporting of outcomes
☒	☐	Estimates of effect sizes (e.g. Cohen's *d*, Pearson's *r*), indicating how they were calculated

Our web collection on statistics for biologists contains articles on many of the points above.

Software and code

Policy information about availability of computer code

Data collection	NMR: Bruker Topspin 4.0 / Bruker IconNMR 5.0.11 Build 10 (part of topspin version 4.0) / Calorimetry: TC Run (v 3.6.5) by TA instruments, Confocal Microscopy: Zen 2.3 SP1 FP3(black) (v14.0.19.201) / HS-AFM: RIBM software HS-AFM 1.0
Data analysis	iNMR version 6.1.9 / NMR: Bruker Topspin 4.0, pymol version 1.3, Calorimetry: NanoAnalyze (version 3.10.0) by TA instruments, Confocal Microscopy: ImageJ (v 2.0.0), HS-AFM: Igor Pro (Wavemetrics, version 9) with RIBM scripts, ImageJ

For manuscripts utilizing custom algorithms or software that are central to the research but not yet described in published literature, software must be made available to editors and reviewers. We strongly encourage code deposition in a community repository (e.g. GitHub). See the Nature Portfolio guidelines for submitting code & software for further information.

Data

Policy information about availability of data

All manuscripts must include a data availability statement. This statement should provide the following information, where applicable:

- Accession codes, unique identifiers, or web links for publicly available datasets
- A description of any restrictions on data availability
- For clinical datasets or third party data, please ensure that the statement adheres to our policy

The NMR assignments of the plectasin – Lipid II complex in different membrane environments have been deposited in the BMRB database (accession number 51880). Experimental solid-state NMR raw data have been deposited in an open repository (DOI: 10.5281/zenodo.7985263).

Research involving human participants, their data, or biological material

Policy information about studies with human participants or human data. See also policy information about sex, gender (identity/presentation), and sexual orientation and race, ethnicity and racism.

Reporting on sex and gender	N/A
Reporting on race, ethnicity, or other socially relevant groupings	N/A
Population characteristics	N/A
Recruitment	N/A
Ethics oversight	N/A

Note that full information on the approval of the study protocol must also be provided in the manuscript.

Field-specific reporting

Please select the one below that is the best fit for your research. If you are not sure, read the appropriate sections before making your selection.

☒ Life sciences ☐ Behavioural & social sciences ☐ Ecological, evolutionary & environmental sciences

For a reference copy of the document with all sections, see nature.com/documents/nr-reporting-summary-flat.pdf

Life sciences study design

All studies must disclose on these points even when the disclosure is negative.

Sample size	ITC experiments were conducted in triplicates. For NMR experiments, experiments were conducted with different samples for 3.2 mm and 1.3 mm rotors, which yielded complementary results.
Data exclusions	MIC values: in case of ambiguity (inconsistent triplicates/controls), assay was repeated and ambigue data were excluded.
Replication	MIC determinations were carried out as technical triplicates, with appropriate controls (both positive and negative) on each individual 96 well plate. Most important analogues were assayed at least twice in triplicate. Consistency of data was checked by taking plectasin (WT) and NZ2114 along as control/reference in each experiment.<br>MIC determination and growth analysis in variable Ca2+ concentrations were assayed in technical duplicates, in two independent experiments.<br><br>For ITC data, three replicates were measured for each experimental setup.<br><br>Attempts to replicate data were successful
Randomization	This is not applicable to the type of experiments and data that we acquire
Blinding	This is not applicable to the type of experiments and data that we acquire

Reporting for specific materials, systems and methods

We require information from authors about some types of materials, experimental systems and methods used in many studies. Here, indicate whether each material, system or method listed is relevant to your study. If you are not sure if a list item applies to your research, read the appropriate section before selecting a response.

Materials & experimental systems

n/a	Involved in the study
☒	☐ Antibodies
☒	☐ Eukaryotic cell lines
☒	☐ Palaeontology and archaeology
☒	☐ Animals and other organisms
☒	☐ Clinical data
☒	☐ Dual use research of concern
☒	☐ Plants

Methods

n/a	Involved in the study
☒	☐ ChIP-seq
☒	☐ Flow cytometry
☒	☐ MRI-based neuroimaging
